# Convpaint—Interactive pixel classification using pretrained neural networks

**DOI:** 10.1016/j.crmeth.2026.101335

**Published:** 2026-03-16

**Authors:** Lucien Hinderling, Roman Schwob, Guillaume Witz, Ana Stojiljković, Maciej Dobrzyński, Mykhailo Vladymyrov, Joël Frei, Benjamin Grädel, Agne Frismantiene, Olivier Pertz

**Affiliations:** 1 Institute of Cell Biology, University of Bern, Baltzerstrasse 4, 3012 Bern, Switzerland; 2 Graduate School for Cellular and Biomedical Sciences, University of Bern, Bern, Switzerland; 3 Data Science Lab, University of Bern, Sidlerstrasse 5, 3012 Bern, Switzerland

**Keywords:** image analysis, pixel classification, multi-dimensional data

## Abstract

We present Convpaint, a universal computational framework for interactive pixel classification. Convpaint uses pretrained convolutional neural networks (CNNs), vision transformers (ViTs), or classical filter banks for feature extraction in combination with fast-to-train machine learning (ML) classifiers to enable easy segmentation across a wide variety of tasks. By integrating ViT-based features, Convpaint extends traditional pixel classification to image domains that require rich semantic understanding. Convpaint’s modular design allows users to rapidly switch between feature extractors, balancing speed, spatial accuracy, and semantic depth based on the specific dataset. Available within the Python-based napari software ecosystem, Convpaint integrates seamlessly with other plugins into image processing pipelines, which we demonstrate with example workflows across different data modalities, from subcellular to cellular to animal scale.

## Highlights

• Convpaint enables rapid pixel classification using pretrained neural networks
• Vision transformers extend segmentation to tasks requiring semantic understanding
• Supports multichannel, 3D, and time-series data
• Integrates into napari for interactive, code-free bioimage analysis workflows

## Motivation

We needed to perform real-time segmentation during feedback-control microscopy experiments, which requires rapid model training on unseen data before starting the experiment. We also required seamless integration into our Python-based microscope control pipeline. Existing solutions fell short: classical interactive machine learning approaches train quickly but lack semantic understanding for complex tasks, while deep learning methods require lengthy training on large datasets acquired previously. This motivated us to develop Convpaint, bridging both approaches to enable accurate segmentation with minimal annotations and fast training times. The method proved so effective that we began applying it to other image analysis tasks beyond feedback microscopy, which prompted us to develop a user-friendly interface and share it with the broader scientific community.

## Graphical abstract

**Figure:**
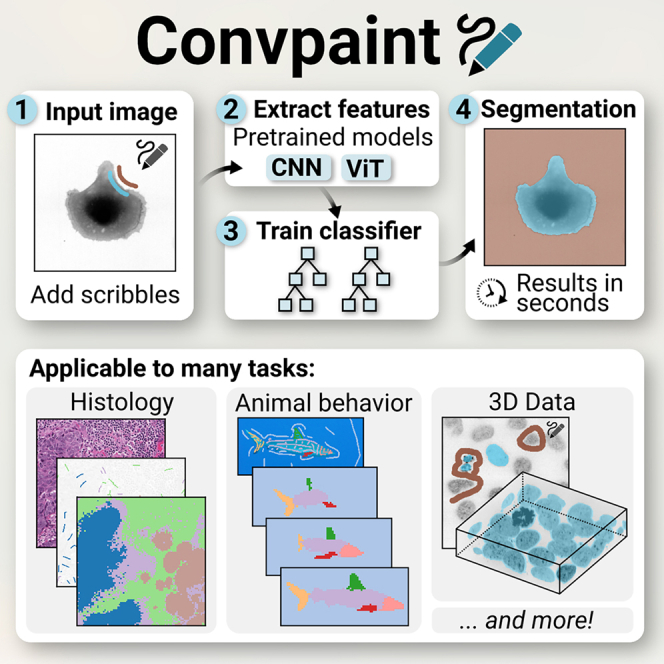

## Teaser

Hinderling et al. present Convpaint, a napari plugin that repurposes pretrained deep learning models for interactive pixel classification. By combining convolutional neural networks and vision transformers with fast machine learning classifiers, Convpaint enables accurate segmentation across diverse imaging modalities with minimal annotations and rapid training times.

## Introduction

Many bioimage analysis pipelines start with a segmentation step. While deep learning (DL) methods offer high classification accuracy, they require extensive ground truth annotation data and can take hours or days to train, even on dedicated hardware.^1^^,^^2^^,^^3^ More recently, self-supervised learning approaches have made it possible to train models without annotated ground truth,^4^^,^^5^ thereby reducing the dependency on manual labeling. However, even foundation models, which are trained on large, diverse datasets and expected to generalize to new applications without retraining, in practice require adaptation for many basic research purposes.^6^^,^^7^^,^^8^^,^^9^ In contrast, machine learning (ML) approaches using small models that can be trained interactively with sparse annotations have proven to be highly effective (e.g., ilastik, Trainable Weka, Qupath, and APOC).^10^^,^^11^^,^^12^^,^^13^ These approaches traditionally rely on hand-crafted filter banks to extract image features and train an ML model from sparse annotations and corresponding features, to predict the class of each pixel in the rest of the image or new images. While these models are quick to train and hand-crafted filter banks effectively describe texture or local image structures,^14^ breakthrough performance in capturing semantically meaningful information from images has been achieved through automatically learned filter banks, specifically convolutional filters in DL models^15^ (for a visual comparison, see Figure S1A). These learned filter banks have been used as feature extractors in combination with classifiers like random forests, support vector machines (SVMs), or XGBoost to quickly adapt to new domains like tomography, satellite images, or textile patterns with little training data and without retraining the DL model.^16^^,^^17^^,^^18^^,^^19^ More recently, vision transformers (ViTs) have surpassed convolutional neural networks (CNNs) in many visual recognition tasks, owing to their ability to model long-range dependencies and global context more effectively.^20^^,^^21^ Convpaint adapts these approaches to pixel-level classification, and makes them accessible in a user-friendly, interactive tool. By combining ML models that are fast to train with the power of DL, Convpaint strikes a balance between training speed, accuracy, and steerability.

## Results

### Convpaint architecture

**Figure 1:**
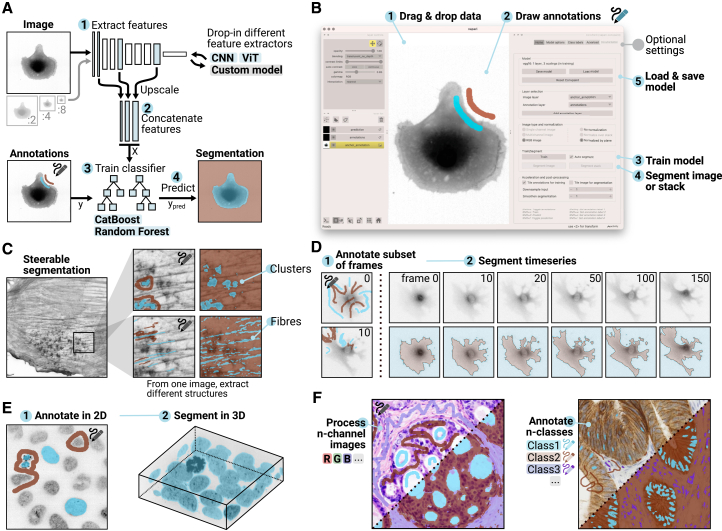
Overview of the Convpaint algorithm, user interface, and capabilities (A) Convpaint architecture: (1) Features are extracted from multiple scalings of the input image using a pretrained neural network. (2) Extracted features are upscaled and concatenated. (3) A classifier is trained on sparse annotations. (4) Prediction of the class for each pixel. Different feature extractor models and classifiers can be used. (B) User interface in napari: (1) Supports various input formats. (2) Annotations are drawn using the label layer. (3) Single-click model training. (4) Single-click image segmentation, with results displayed in a label layer. (5) Model is saved for future use. Optional settings allow choosing image preprocessing, feature extractor parameters, and acceleration options like GPU or parallel processing. (C) User annotations interactively guide segmentation, allowing different structures to be extracted from the same image. (D) Segments time-series data across all frames with a single click, enabling immediate playback. Fine details like cellular protrusions can be accurately segmented. (E) Use of napari’s visualization to verify 3D segmentation results. (F) Adapts to any number of input channels and output classes.

Convpaint enables rapid repurposing of pretrained DL models for new tasks without retraining. By leveraging pretrained models, such as CNNs or ViTs, Convpaint extracts local image features and combines them with lightweight classifiers like CatBoost^22^ or random forests^23^ (Figure 1A). Integrated into the napari ecosystem,^24^ Convpaint offers a graphical user interface (GUI) that allows researchers to train models for specific tasks without requiring coding or machine learning expertise (Figure 1B). For advanced users, Python application programming interfaces (APIs) provide a programmatic control, with tutorials available in the documentation for integrating Convpaint into Jupyter notebooks and the scientific Python ecosystem.

First, we use a CNN (VGG16) pretrained on general image data as a feature extractor.^25^ Unlike neural networks trained for predefined tasks, such as detecting specific structures (e.g., spots or fibers), Convpaint enables users to guide the model through sparse annotations on regions of interest. For example, as shown in Figure 1C, different segmentations of polymerized actin clusters and stress fibers in fibroblasts expressing an actin filament marker can be obtained from the same image simply by annotating different structures. Using VGG16 as a feature extractor, Convpaint generalizes across diverse image domains, from microscopy to photos of objects and natural scenes (additional examples in Figure S1B). Convpaint seamlessly processes multi-dimensional data, making it well suited for segmenting time series and 3D datasets. As depicted in Figure 1D, Convpaint captures fine protrusions and jagged edges in a time-lapse video of a migrating fibroblast cell, while Figure 1E shows segmentation of nuclei in a 3D stack. Convpaint supports training with any number of input channels and output classes, as demonstrated with histology data in Figure 1F and further elaborated using synthetic data in Figures S1C–S1E.

Video S1 showcases Convpaint’s speed and interactivity in a 3D segmentation task. The entire workflow, from annotating a z stack to verifying results on another slice, correcting misclassified pixels, updating the model, segmenting the full stack, and visualizing the results in 3D, is completed in 10 s on a laptop (2021 MacBook Pro M1 Max with MPS disabled, 20 s on 2021 HP Elitebook 850 G8 HP). This rapid feedback loop allows users to quickly refine segmentation quality and intuitively interpret results, even on complex multidimensional datasets. Convpaint allows to repurpose pretrained DL models to new image analysis tasks within minutes by using them as feature extractors. As an illustrative example, intermediate outputs of a Cellpose U-net model, originally trained to predict cell masks, can be used with Convpaint to segment other structures such as cell boundaries, nuclei, or mitotic cells with minimal annotations (Figure S1F). Convpaint’s modular design supports swapping feature extractors and classifiers to accommodate specific needs, which we will discuss in detail later in the paper. This modularity also ensures Convpaint can incorporate the latest advances in computer vision.

Video S1. Screen recording of training Convpaint to segment a 3D stack, related to Figure 1

Several architectural optimizations enhance Convpaint’s efficiency during training and prediction. The system extracts crops around annotated pixels, minimizing unnecessary processing of entire images. Iterative annotations are computed lazily; when an annotation is added in a different plane or tile, Convpaint calculates features only for that region, instead of reprocessing the entire image; Dask integration enables handling of data that exceed available memory. For large images and image stacks, tiled parallel processing substantially reduces both memory footprint and processing time. In a sample dataset of 14-megapixel images,^26^ we observed more than a 5-fold reduction in both memory usage and computation time (see Figures S2A and S2B). Built-in options for downsampling and upsampling, image normalization either per plane or across the full stack, and post-processing of labels are directly accessible from the GUI. Users can also switch between different feature extractors and store or load classifiers directly from the GUI. All of these optimizations and usability features remain fully supported, even when custom feature extractors or classifiers are added. For users interested in integrating their own feature extractor, we provide a well-documented blueprint to make it easy to get started with development.

### Example workflows

Convpaint integrates seamlessly with various napari plugins, enabling complex image analysis workflows within a single software ecosystem without requiring coding. We demonstrate this capability through three workflows, again using the CNN VGG16 as feature extractor.

**Figure 2:**
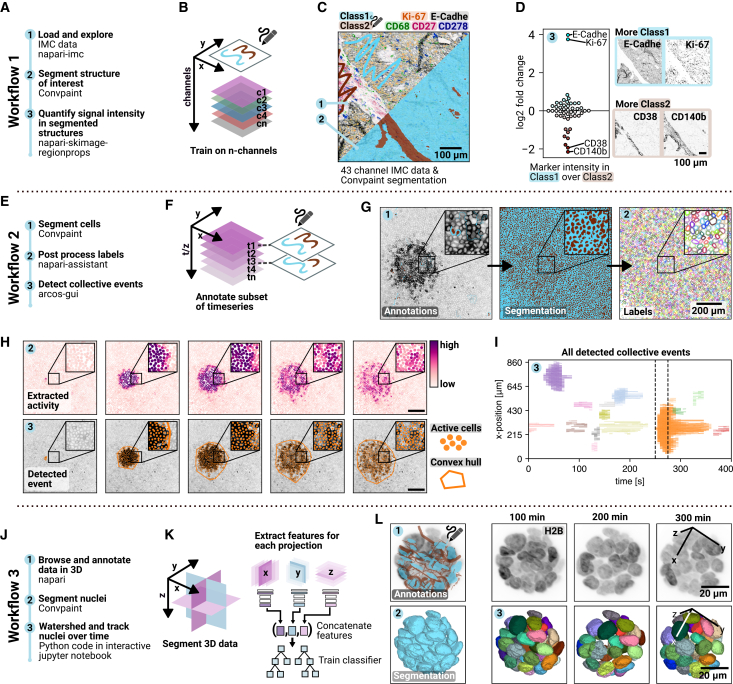
Image analysis workflows using Convpaint Workflow 1 (multichannel dataset): (A) Example with multichannel IMC data. (B) Handles arbitrary input channels. (C) Interactive exploration with napari-imc. Labeled structures guide segmentation across all channels. Scale bars: 100 *μm*. (D) Use class labels for data exploration and statistical analysis, such as identifying differentially expressed markers. Scale bars: 100 *μm* m. Workflow 2 (time-series): Supports 3D and time-series data. (E) Combined with arcos-gui to detect collective signaling events in MDCK cell videos. (F) Train classifier on a subset of frames or z slices to predict the whole stack. (G) Segmentation of nuclei with Convpaint; post-processing and instance segmentation with napari-assistant. Scale bars: 20 *μm*. (H) Example of calcium wave activation across five frames, with quantified signaling activity and detection of collective behavior shown as an overlay. Scale bars: 200 *μm*. (I) Overview of all detected events, with the period from (H) marked. See full data in Video S2. Workflow 3 (3D segmentation): (J) Segmentation of MCF10A acini from lightsheet microscopy, with 3D watershed instance segmentation and tracking with trackpy. (K) Feature extraction from multiple projections (*xy*, *xz*, and *yz*) combined for random forest classification. (L) 3D rendering of tracked nuclei with color-coded IDs. Scale bars: 20 *μm*. See full data in Video S3.

Workflow 1 showcases Convpaint’s ability to handle multichannel data, a common challenge in imaging mass cytometry (IMC), spatial transcriptomics, or multiplexed immunofluorescence imaging, where numerous biomarkers need to be visualized in the same sample. These techniques generate rich datasets, posing challenges for interactive exploration and analysis. In Figures 2A–2D, we present an example using a 43-channel IMC dataset.^27^ The data are interactively loaded and browsed with the napari-imc plugin.^28^ Instead of exporting data for pixel classification to an external software such as ilastik, as shown in a workflow of a previous study,^28^ Convpaint can perform segmentation directly within napari. Scribbles are used to segment veins and surrounding tissue regions, and differentially expressed markers between the two classes are identified. CD38 and CD140b (PDGFR*β*) are mostly abundant in the veins and mark immune cells and pericytes,^29^ respectively. In the tissue surrounding the veins, the adhesion molecule E-cadherin marking epithelial cells and the cell proliferation marker Ki-67 show the highest fold change. This workflow would allow a pathologist to quickly mark structures of interest in an field of view (FOV), find regions with similar tissue structure in the rest of the slide, and extract information like proliferation marker abundance to quantitatively compare tumor aggressiveness.

Workflow 2 demonstrates Convpaint’s ability to analyze time-lapse data with interactive visualization in napari, applied to detect collective calcium signaling waves in an epithelial monolayer expressing a calcium biosensor (Figures 2E–2I and Video S2).^30^ First, Convpaint is trained with scribbles across multiple frames to segment all nuclei. The resulting labels are post-processed using another napari plugin, napari-assistant,^31^ which performs instance segmentation and extracts biosensor intensity time-series data for each individual cell. Finally, calcium waves are identified by calculating spatiotemporal correlations in signaling activity using the ARCOS plugin,^32^^,^^33^^,^^34^ a computational method to detect and quantify collective phenomena.

Video S2. Detection of calcium waves in MDCK cells, related to Figure 2

In Workflow 3, we segment and track nuclei in a lightsheet time-lapse dataset of 3D mammary acini. To enhance segmentation in 3D datasets, Convpaint combines features extracted from *xy*, *xz*, and *yz* projections, which are concatenated as inputs to a random forest classifier (Figure S2C illustrates that incorporating multiple projections improves segmentation on synthetic 3D data). The mammary acini in this workflow are MCF10A cells expressing a histone H2B nuclear marker and ERK-KTR, a biosensor reporting ERK activity via nucleus-cytosol translocation following phosphorylation by active ERK^35^ (scheme in Figure S3A). After segmentation with Convpaint, a 3D watershed algorithm is applied for instance segmentation, followed by nuclei tracking using an overlap-based algorithm (Video S3). In Figures S3B–S3F, we demonstrate how Convpaint enables tracking of individual cells within dense spheroids and extraction of single-cell ERK signaling activity over time. The analysis reveals pulsatory ERK dynamics, consistent with previous findings.^35^

Video S3. Segmentation of MCF10A acini, related to Figure 2

### Using ViTs for feature extraction

**Figure 3:**
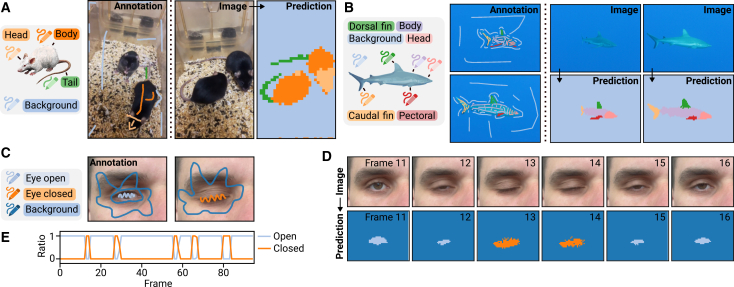
Using DINOv2 as feature extractor in Convpaint (A) When coupled with different feature extraction models, Convpaint can be applied to bioimages across scales, from subcellular to cellular structures in animals. Here, we train Convpaint to detect mouse body parts on 4 frames (one shown) and use it to predict the rest of the video (see Video S4 for full data). (B) Convpaint is trained to detect shark body parts in a video (2 out of 3 annotated frames are shown). Prediction on two unseen frames are shown (see Video S4 for full data). (C) Convpaint can be applied to behavior detection; here, it is trained to distinguish between open and closed eyes, using two annotated frames. (D) Sample frames from the video showing a blink event (see Video S5 for full data). (E) Quantification of the segmentation detects multiple blink events in the video (y axis shows the ratio of pixels classified as open versus closed per frame).

Until now, all results in this paper have used the first layers of a CNN (VGG16) as the feature extractor. However, as mentioned earlier, other DL models can be repurposed with Convpaint for different image segmentation tasks in minutes. During development, we found that ViTs, which extract semantically richer features, significantly improve segmentation performance, particularly on complex datasets that challenge traditional pixel classifiers based on classical filter banks. Although Convpaint was initially designed for microscopy image segmentation, the integration of ViTs has broadened its applicability to tasks unattainable with conventional pixel classifiers. Using the ViT DINOv2, pretrained on a large, diverse image dataset,^4^ Convpaint can segment mouse body parts in a video using annotations from just a few frames (Figure 3A and Video S4). This approach is applicable across species: Figure 3B and Video S4 also demonstrate Convpaint’s ability to segment different body parts, and even fin types in a shark. Beyond anatomical labeling, Convpaint can annotate behaviors, such as distinguishing between open and closed eyes. With scribbles from only two frames, one showing an open eye and another a closed eye, Convpaint detects human blinking events in a video (Figures 3C and 3D; Video S5). Eye state classification also generalizes to rats (Video S5). These examples demonstrate that Convpaint’s interactive segmentation capabilities extend beyond traditional biological imaging and can support research in diverse fields such as behavioral neuroscience, ecology, ethology, and other domains involving complex visual data.

Video S4. Segmentation of animal body parts, related to Figure 3

Video S5. Detection of open/closed eyes in humans and mice, related to Figure 3

Although DINOv2 captures strong semantic information, its patch-based architecture extracts features at a resolution of 14 × 14 pixels, limiting spatial detail and causing fine structures and boundaries to be lost, which reduces pixel-level segmentation accuracy. Testing Convpaint with different feature extractors across multiple data types and inspecting the resulting feature representations, we observed complementary strengths among the models: ViTs are particularly effective at capturing abstract semantic information at the patch level (e.g., head, tail, or cancerous tissue), whereas CNNs and classical filters extract precise local structural information (e.g., textures, edges, or colors) with high spatial resolution. This observation motivated us to combine the pixel-accurate segmentation of VGG16 with the semantic understanding of DINOv2 (Figure S4A) by simply concatenating their features. For example, while DINOv2 reliably differentiates shark body parts, its patch size limitations reduce spatial precision. Conversely, VGG16 accurately masks the shark but lacks the semantic understanding to label anatomical structures correctly. Combining the outputs of both models achieves semantically correct labels with high spatial resolution (note that we used the DINOv2 variants with registers, as these produced less patch noise in the predictions, Figure S4B). Recently, new approaches have emerged to increase the spatial resolution of feature representations.^36^^,^^37^ Models such as the JAFAR upscaler^38^ are trained to refine the patch-sized features of transformer-based models, using the input image as additional guidance. We also integrated JAFAR into Convpaint to enhance the pixel-level detail of the DINOv2 model. Given the growing diversity of implemented feature extractors, we next sought to systematically evaluate their respective strengths and limitations across different datasets and segmentation tasks.

### Quantification of feature extractor performance across diverse datasets

**Figure 4:**
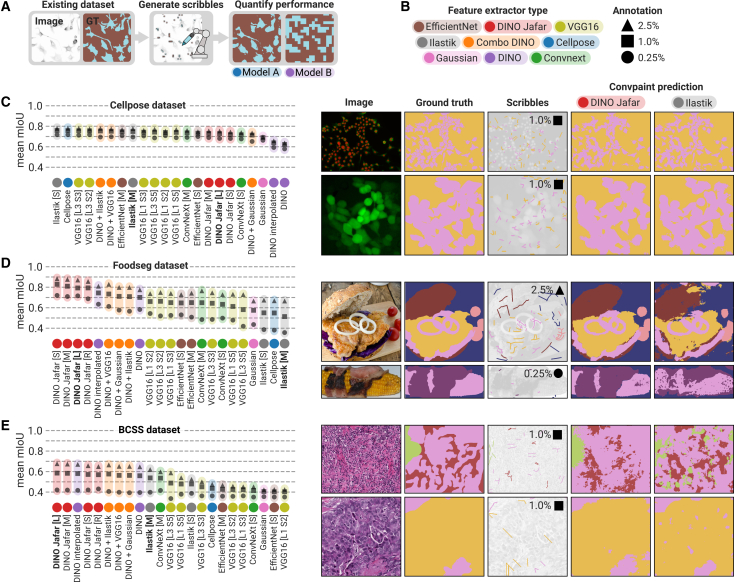
Segmentation performance of different feature extractors (A) Different publicly available segmentation datasets were used to automatically generate scribble annotations from ground truth for training Convpaint with various feature extraction models. Segmentation performance was evaluated against the ground truth, using the mean intersection over union (mIoU) metric. (B) A total of 23 feature extractors were tested, grouped into nine categories. See Table S1 for a detailed description of all models. We also assessed performance at different annotation levels, corresponding to 0.25%, 1.0%, or 2.5% of all image pixels being annotated. In total, we evaluated *n* = 108,642 combinations of annotations, models, and images. Among these, two representative models are highlighted throughout the figure: the ilastik feature extractor, which implements a classical filter-bank approach expected to perform well on data that can be segmented using low-level local image information (such as pixel intensity, edges, texture, and local contrast), and the DINOv2 + JAFAR model, which uses a vision transformer pretrained on general image data to capture more semantic and global image information, combined with an upscaler to increase the patch-level resolution of the vision transformer outputs, enabling pixel-level segmentation predictions. (C) Results on the Cellpose dataset (*n* = 540 images tested per model per annotation level, instance labels were converted to semantic labels). This dataset represents fine-scale foreground/background segmentation tasks that rely on low-level image cues. Most classifiers achieved comparable mIoU scores, except for DINOv2-only models, which were limited by their large patch size and inability to capture small cellular details. The input image, ground-truth mask, generated scribbles (expanded for visualization), and segmentation outputs for the two representative feature extractors (DINOv2 + JAFAR upscaler and the ilastik filter set model) are shown in the right. (D) Results on the FoodSeg dataset (*n* = 520 images tested per model per annotation level), used to evaluate segmentation on natural images. Unlike the Cellpose dataset, which emphasizes low-level image features, FoodSeg is expected to test models’ ability to extract more semantic feature information and global context. Models based on DINOv2 backbones, particularly the DINOv2 + JAFAR upscaler variants, achieved the highest mIoU. (E) Results on an expert-annotated histology dataset (*n* = 538 images tested per model per annotation level). Similar trends to the FoodSeg results were observed, indicating that natural image feature extractors generalize to certain medical imaging domains. Sample images showing segmentation performance of all feature extractors on the different datasets are provided in Data S1.

To address the lack of standard ground-truth test datasets for evaluating interactive pixel classifiers, we developed a computational pipeline that generates test datasets for Convpaint. The pipeline creates human-like scribbles from existing datasets with full-image ground truth, enabling unbiased quantitative assessments of Convpaint’s segmentation performance across datasets (Figure 4A).

We applied this approach to three segmentation datasets that differ in the types of visual features relevant for accurate segmentation: (1) the Cellpose dataset,^1^ capturing diverse cellular morphologies across fluorescence and bright-field modalities; (2) FoodSeg103,^39^ comprising complex natural images of dishes with ingredient-level semantic annotations; and (3) Breast Cancer Semantic Segmentation (BCSS) dataset with expert-annotated tissue types.^40^ For each dataset, Convpaint was trained on simulated scribble annotations derived from the ground truth and then used to predict the remaining unlabeled regions. Prediction accuracy was quantified using the mean intersection over union (mIoU) metric across multiple annotation densities (0.25%, 1.0%, and 2.5% of annotated pixels).

We evaluated a range of feature extractors, beginning with CNN-based architectures including EfficientNet,^41^ ConvNeXt,^42^ VGG16,^25^ and a U-Net trained on one of the tested datasets (Cellpose3^43^), which we included to assess how pretraining on a domain-specific dataset compares to general pretraining on large-scale, natural image data. We further benchmarked the classical filter bank approach (ilastik,^10^ using filters as implemented in ilastik-napari^44^) and a simple Gaussian filter used as a baseline. Finally, we tested ViT-based models, including standalone DINOv2^4^ and DINOv2 combined with other feature extractors or the upscaler JAFAR^38^ to compensate for the coarse, patch-level features produced by DINOv2. Multiple configurations of these models were explored; a complete overview is provided in Table S1, and detailed implementation descriptions are presented in the STAR Methods section. For clarity and ease of comparison, we grouped the models into nine color-coded categories consistently used throughout the figures in this section (Figure 4B).

In the Cellpose dataset (Figure 4C), segmentation relies mainly on local image features such as intensity, texture, and edge information. Accordingly, classical filter banks (ilastik) and CNN-based models like VGG16 performed well, capturing fine structural details such as cell boundaries and protrusions. Using the Cellpose model as a feature extractor that was pretrained on this dataset yielded similar performance to CNNs pretrained on general image data, suggesting that domain-specific pretraining offers only limited improvements in this context. DINOv2 was constrained by its patch-level resolution, leading to poor accuracy on small-scale details such as fine cellular protrusions. Incorporating the JAFAR upscaler or combining DINOv2 features with those from other models, even a simple Gaussian filter, resulted in noticeable performance improvements. Increasing the annotations led to little improvements across models, indicating that the performance score is limited by other factors, in some cases by the quality of ground-truth annotations (see Figure S4C). Note that the Cellpose dataset was originally designed for instance segmentation, but for the purposes of this study, we converted it into a simpler foreground-background segmentation task.

The FoodSeg dataset (Figure 4D), by contrast, requires higher-level semantic understanding and the integration of global image context to separate visually similar objects (here meal ingredients) in natural images. For instance, bread and grilled chicken may exhibit similar textures and colors at small scales, making it necessary to consider the broader scene (the structure of the sandwich) to classify them accurately. Here, transformer-based feature extractors perform best, benefiting from their ability to encode large-scale spatial relationships and contextual cues, followed by CNNs. Increasing the annotation fraction improved performance across all models, most notably for those relying on low-level feature representations such as ilastik.

Finally, the BCSS histology dataset (Figure 4E) combines both local and global challenges, requiring sensitivity to fine textures as well as contextual interpretation of tissue organization. On this dataset, models with a DINOv2 backbone again achieved the highest accuracy. For this dataset, increasing the number of annotations did not substantially improve the performance of weaker models, suggesting that their feature representation capacity (rather than annotation density) is the primary limiting factor. We additionally tested DINOv2 versus UNI,^7^ a model with the same transformer architecture as DINOv2 but specifically trained on histology data. On a subset of the BCSS dataset, DINOv2 achieved significantly higher mIoU scores (Figures S4D and S4E). These results underscore the strong generalization capability of models pretrained on large and diverse image datasets, even to medical domains.

**Figure 5:**
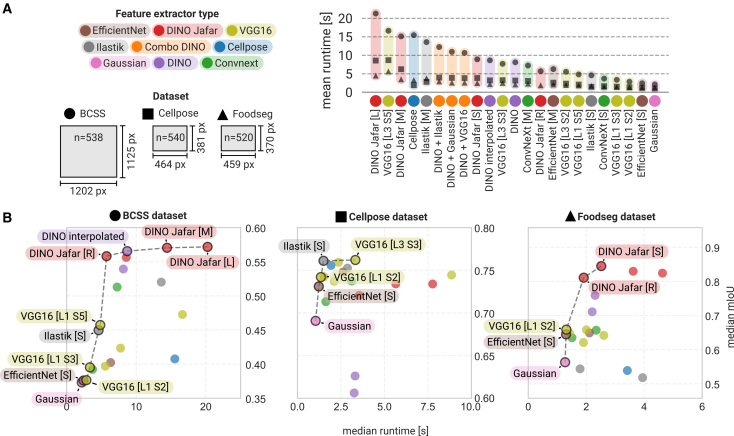
Trade-off between segmentation performance and runtime (A) Runtime measurements (training plus prediction) for different feature extractors across the three datasets. The schematic indicates the mean image dimensions per dataset. Runtime differences become particularly evident for large images in the BCSS dataset. All tests were performed with tiling disabled. (B) Plotting of runtime against the mIoU score. Dashed line marks Pareto front, the best models for a certain runtime/mIoU trade-off. Note that scales are different between the subplots. There is no single model that could achieve the best segmentation performance across all datasets, and within each dataset, different feature extractors offer distinct trade-offs between accuracy and speed. This underscores the value of being able to rapidly test and switch between models, a process made straightforward in the Convpaint GUI.
• The software is open source (BSD-3) and hosted on GitHub^60^ (archived on Zenodo^61^), with installation options available via the napari hub or PyPI. Convpaint supports all major operating systems and runs on standard consumer hardware, with optional GPU acceleration. Installation instructions, documentation, and video tutorials are available online.^62^
• Any additional information required to reanalyze the data reported in this paper is available from the lead contact upon request.

In addition to segmentation accuracy, we evaluated the computational efficiency of each feature extractor (Figure 5A). Runtime analyses (feature extraction, training + prediction) revealed substantial variation across models and datasets, with transformer-based approaches being more computationally demanding than CNNs or filter-based methods. This is noticeable, especially for larger images, as self-attention of ViTs scales quadratically with the input image size.^45^ Plotting runtime against mIoU scores (Figure 5B) highlights the trade-off between segmentation performance and processing speed: no single model could achieve optimal results across all datasets, and each feature extractor offered a distinct balance between accuracy and efficiency. A practical way to retain high processing speeds on the BCSS dataset with ViTs was to downscale the input image by a factor of two before feature extraction and then smooth the upscaled prediction with a majority filter (model *DINO Jafar [R]*), which resulted in only a minor drop in mIoU. Conveniently, such pre- and post-processing options can be configured and tested directly within the Convpaint GUI for any classifier.

Together, these results show that segmentation performance depends strongly on the type of visual information most relevant to each dataset, and there is no single best classifier. The ability to rapidly experiment with different feature extractors, combinations of feature extractors with complementary strengths, and processing strategies in Convpaint provides a clear, practical advantage, enabling users to efficiently identify the configuration best suited to their dataset’s structure, scale, and computational constraints. At the same time, this breadth of available options can feel overwhelming; hence, we provide an accessible guide in the online documentation that explains the different models, highlights where each tends to perform well, and outlines the key trade-offs to be considered. The guide also includes practical tips and annotation strategies to help users achieve the best results with minimal manual effort.

Scores for all models are provided in Table S1, and additional randomly sampled segmentation examples are shown in Data S1. The complete pipeline for generating test data and reproducing these analyses is available on GitHub.^46^

## Discussion

Convpaint was originally developed in our lab to address a specific challenge in feedback control microscopy—the need for real-time cell segmentation during live experiments.^47^ In these scenarios, the appearance of cells is often unpredictable, making it impractical to rely on pretrained models or train models after data collection. Instead, we required a tool that could be quickly trained on live data directly at the microscope. At the time, no existing tools could integrate seamlessly into our Python-based microscope control pipeline. Since we were already using napari for visualizing the camera feed, we developed Convpaint as a napari plugin. This allowed us to automatically detect subcellular regions for targeted photoactivation, leading to new insights into the spatiotemporal regulation of RhoGTPase signaling.^48^ Convpaint’s success in these experiments quickly led us to adopt it for other lab projects. Its broad applicability ultimately encouraged us to share it with the broader scientific community.

We show that by combining pretrained DL models with fast-to-train ML classifiers, we can quickly adapt them for pixel classification across diverse domains. In particular, by leveraging the patch-level semantic features of ViTs, we can significantly improve segmentation performance on tasks that require high-level contextual understanding. Traditional feature extractors like classical filter banks struggle with these tasks because they rely primarily on local image features such as intensity, texture, or edges. These low-level cues are not sufficient when the visual distinctions between classes are subtle or ambiguous, for example, in cases like segmenting animal body parts across variable poses and lighting conditions, where semantic context is essential. In contrast, ViT-based models can incorporate global image context, enabling Convpaint to generalize to challenging use cases that are typically beyond the capabilities of classical pixel classifiers. In our experiments, we found that different datasets tend to favor different combinations of feature extractors and classifiers, with no single model performing the best across all cases. The ability to choose between different feature extractors allows users to tailor the approach to their specific data characteristics and computational constraints, balancing accuracy, speed, and resource efficiency.

Since the preprint of this paper, other groups have explored similar approaches. Weakly supervised segmentation using patch-based DINOv2 features with random forests has been reported,^36^ while a related project^49^ employs a different vision model.^50^ CellCanvas^51^ introduced a transformer-based feature extractor^52^ combined with an XGBoost classifier^53^ for 3D electron microscopy data. The ViT was pretrained self-supervised on medical images and subsequently refined on synthetic data, pointing toward a future where transformer models can be trained on custom datasets without ground-truth annotations. In contrast, our results indicate that broadly pretrained models can outperform domain-specific ones (DINOv2 vs. UNI, Figures S4D and S4E). It will be interesting to see how the trade-off between smaller, domain-specific models and broadly pretrained models plays out in practice as these approaches continue to mature. In general, these developments underscore a growing trend in adapting large vision models for interactive segmentation tools. As discussed, many datasets can still be effectively segmented using classical filter banks. For such cases, APOC^13^ provides an alternative napari plugin that offers a fast, GPU-accelerated solution for pixel and object classification. In contrast to Convpaint, APOC natively supports object-level classification, and the two tools can be combined within the same workflow, using Convpaint for pixel classification and APOC for subsequent object classification (via napari-assistant, see Workflow 2 in Figure 2). This complementarity highlights the strength of an open, modular ecosystem, where napari plugins interoperate seamlessly across different image analysis tasks and experimental setups.

Within this ecosystem, Convpaint contributes a flexible and accessible framework for advanced feature extraction and pixel-level classification. Its feature-rich graphical interface allows users to interactively train models and visualize results without programming, lowering the barrier for applying advanced image analysis methods. Through its API, Convpaint can be integrated into automated pipelines and used for batch processing, for example in feedback-control microscopy. Its modular architecture supports the addition of new feature extractors or processing modules, enabling rapid adaptation to emerging methods in computer vision. In this way, Convpaint bridges usability and flexibility, helping to make state-of-the-art computer vision methods broadly applicable across biology.

### Limitations of the study

Convpaint relies on pretrained models or filter banks, meaning its performance is bounded by the feature representations these models provide. While ViTs like DINOv2 capture rich semantic information, their patch-based architecture limits spatial resolution, causing fine structures and boundaries to be lost. Although combining ViT features with CNN features or upscalers like JAFAR partially addresses this, transformer-based feature extractors remain computationally demanding, with processing time scaling quadratically with image size. This creates a trade-off between accuracy and computational efficiency that users must navigate. Finally, there is no single best feature extractor across all datasets. Users must experiment and develop an intuition for which model works best for their specific task, which can feel overwhelming despite our documentation and guidance.

## Resource availability

### Lead contact

Requests for further information and resources should be directed to and will be fulfilled by the lead contact, Olivier Pertz (olivier.pertz@unibe.ch).

### Materials availability

This study did not generate new materials.

### Data and code availability

• Workflow 1: IMC multichannel data. IMC data from Eling and Windhager (2022),^27^ available on Zenodo: https://doi.org/10.5281/zenodo.5555575, were loaded using the napari-imc plugin.^28^ Convpaint was trained on one FOV (Figure 2 shows Patient 01, Panorama 02, Position 1-1). Skimage was used to extract per-channel statistics for the segmented regions. The log_2_-fold change in signal intensity was calculated using NumPy and plotted with Matplotlib. Step-by-step instructions for recreating the workflow are available in the documentation (https://guiwitz.github.io/napari-convpaint/book/IMC_data.html). Workflow 2: MDCK calcium waves. Time-lapse datasets of calcium signaling waves were obtained from MDCK epithelial cells that stably express GCaMP6S—a genetically encoded intracellular calcium sensor (imaging data courtesy: Yasuyuki Fujita).^30^ The videos were loaded into napari, and cell nuclei were segmented using Convpaint. The resulting binary masks were processed with ARCOS^32^ to detect and quantify collective signaling events. The code to recreate the figures is available in the documentation (https://guiwitz.github.io/napari-convpaint/book/Calcium_waves.html). We have made the raw imaging data available on the BioImageArchive: S-BIAD1135.^54^ Step-by-step instructions for recreating the workflow are available in the documentation. **W**orkflow 3: 3D segmentation and nuclei tracking. Data were acquired using a lightsheet microscope with a 5-min resolution and an isotropic voxel size of 0.145 *μ*m. Raw imaging data and protocols are available on BioImageArchive: S-BIAD1134.^55^ Other figures. The 3D nuclear data in Figure 1E and Video S1 are part of the scikit-image^56^ data module, called *cells3d*, originally provided by the Allen Institute for Cell Science. The synthetic data in Figure S2C were generated by another group using FiloGen^57^ and are available from the Broad Bioimage Benchmark Collection: BBBC046,^58^ showing cell PD-ID451/AR1/T024. The datasets used for performance quantification shown in Figures 4 and 5, Cellpose,^1^ FoodSeg103,^39^ and the Breast Cancer Semantic Segmentation (BCSS) (https://bcsegmentation.grand-challenge.org/BCSS/),^40^ have all been previously published. Video S4 shown in Figure 3A is a supplement (https://doi.org/10.3389/fgene.2018.00581.s007) to a study on epileptic behavior in mice.^59^ Videos S4 and S5 of shark and human eye in Figures 3B–3D were acquired by the authors and are available upon request. Video S5 of rat is available online (https://mixkit.co/free-stock-video/gray-and-white-rat-32060/) for educational purposes under the Mixkit Restricted License. Images in Figure S1B were acquired by the authors and are available upon request, except for the histology slide images, also shown in Figure 1, which are from Wikimedia Commons (https://commons.wikimedia.org/wiki/File:Breast_DCIS_histopathology_(1).jpg) or provided by the scikit-image library, acquired at the Center for Microscopy And Molecular Imaging (CMMI). The dataset in Figures S2A and S2B is available for academic research under Creative Common Attribution-NonCommercial 2.0 Generic license.^26^

## Acknowledgments

This work has been supported by the 10.13039/100014989Chan Zuckerberg Initiative (CZI) grant NP2-0000000095 to L.H. and O.P., Uniscientia fellowship 187–2021 to O.P., 10.13039/501100001711SNF Sinergia grant CRSII5_183550 to O.P., 10.13039/501100001711Schweizerischer Nationalfonds (SNF) grant 310030_185376 to O.P., and the Digitalization Commission of the 10.13039/100009068University of Bern (DigiK) financial support to L.H. We thank Loïc A. Royer for suggesting the use of CatBoost, which significantly improved the segmentation speed. We thank the Scientific Center for Optical and Electron Microscopy (ScopeM) of ETH Zurich, Switzerland, for providing access to their instruments and services and Dr. Tobias Schwartz for his assistance in acquiring lightsheet data. Calcium imaging data were kindly provided by Yasuto Takeuchi and Yasuyuki Fujita. Other microscopy experiments were performed on equipment supported by the Microscopy Imaging Center (10.13039/100016167MIC, University of Bernn, Switzerland. The mouse icon in Video S4 by DBCLS https://togotv.dbcls.jp/en/pics.html is CC-BY 4.0 licensed.

## Author contributions

L.H. conceptualized the work. L.H., G.W., R.S., A.S., M.D., M.V., and B.G. contributed to the development of the software and documentation. R.S. quantified performance. J.F., L.H., B.G., R.S., and A.F. acquired data. L.H., G.W., and O.P. supervised the work. Figures were created by L.H. L.H. and O.P. wrote the manuscript and acquired funding. All authors read and approved the final manuscript.

## Declaration of interests

The authors declare no competing interests.

## Declaration of generative AI and AI-assisted technologies in the writing process

During the preparation of this work, the authors used ChatGPT (OpenAI) and Claude (Anthropic) for text editing, specifically to improve grammar and sentence structure, but not to generate new scientific or conceptual content. After using these tools, the authors reviewed and edited the content as needed and take full responsibility for the content of the publication.

## STAR★Methods

### Key resources table

**Table:** 

REAGENT or RESOURCE	SOURCE	IDENTIFIER
**Deposited data**		

IMC data	Zenodo	https://doi.org/10.5281/ZENODO.5555575
Calcium signaling wave data	BioImageArchive	S-BIAD1135
Lightsheet acini data	BioImageArchive	S-BIAD1134
Synthetic 3D cell data	Broad Bioimage Benchmark Collectio	BBBC046

**Software and algorithms**		

Convpaint	GitHub and Zenodo	www.github.com/guiwitz/napari-convpaint; https://doi.org/10.5281/ZENODO.18329731
Napari	Zenodo	https://doi.org/10.5281/ZENODO.3555620
napari-assistant	Zenodo	https://doi.org/10.5281/ZENODO.7308235
APOC	Zenodo	https://doi.org/10.5281/ZENODO.10071078
ilastik-napari	GitHub	https://github.com/ilastik/ilastik-napari
napari-imc	GitHub	https://github.com/BodenmillerGroup/napari-imc
arcos4py	Zenodo	https://doi.org/10.5281/ZENODO.17778664

### Method details

#### Convpaint implementation details

Convpaint features a modular architecture designed to accommodate a wide range of feature extractors, enhancing existing algorithms or pretrained models with added steerability. We compare three different types of feature extractors:

**CNNs:** We use the VGG16^25^ architecture implemented in pytorch,^63^ pretrained on the ImageNet dataset,^64^ to extract local image features such as edges, textures, and color channel correlations when working with RGB images. Downscaled versions of the input image are passed through VGG16, creating a featurized image pyramid. These features are then upscaled and concatenated with unscaled outputs and deeper CNN layer features, balancing segmentation speed and accuracy. This method effectively generalizes to a variety of image segmentation tasks (Figure S1B). We evaluated different configurations of input scalings and layers for feature extraction and provide default settings that perform well on all of the tested datasets. Similarly, other CNN architectures available in Convpaint are ConvNext^42^ and EfficientNet,^41^ both are implemented in PyTorch and have model weights available from the PyTorch model zoo (EfficientNet-B0: efficientnet_b0_rwightman-7f5810bc.pth; ConvNeXt (base): convnext_base-6075fbad.pth). Lastly, we added a pretrained Cellpose3 model (residual U-net) as feature extractor,^43^ using the tissuenet_cp3 weights.

**ViTs:** We incorporate two ViT models, DINOv2^4^ and UNI.^7^ DINOv2 is pretrained on 142 M images from ImageNet, while UNI is pretrained on a large histology dataset. These models extract patch features of 14 × 14 pixels (DINOv2) and 16 × 16 pixels (UNI), providing superior performance in certain segmentation tasks despite a loss in resolution for fine details below the patch size, like small cell protrusions. For each patch, the ViT-S/14 distilled DINOv2 model we used extracts 384 features, while UNI extracts 1024 features. As mentioned, for all DINOv2 experiments, we used the variant with registers, as this configuration produced less patch noise in the predictions (Figure S4B). As upscaler for DINOv2, we used JAFAR^38^ with pretrained weights corresponding to the backbone used (named ViT-S-Reg4-14-DINOv2).

**Classical filter bank:** To compare the performance of Convpaint using pretrained neural networks versus classical filter banks as feature extractors, we employed the filters implemented in ilastik-napari (https://github.com/ilastik/ilastik-napari) (v0.2.4). We chose the maximal combination of filters and sigma parameters suggested in the library, including Gaussian, Laplacian of Gaussian, Gaussian gradient magnitude, difference of Gaussians, structure tensor eigenvalues, and Hessian of Gaussian eigenvalues, with sigma values 0.3, 0.7, 1.0, 1.6, 3.5, 5.0, 10.0. Figure S1A shows a visual comparison of filters used in classical filter banks versus learned convolutional filters extracted from VGG16. As a baseline, we also added a simple Gaussian filter (implemented in scikit-image) with sigma = 3.

Convpaint is optimized for both training and prediction efficiency.

• Crop around annotations: Avoid processing entire images by extracting cropping around annotated pixels.
• Tiling and parallel processing: Handle large images by tiling them and using parallel processing, with appropriate padding to minimize edge effects. One-click batch processing for image stacks.
• Data management: Manage larger-than-memory files using Dask, appropriate handling of additional image dimensions (channels vs. time/z-slices)
• Customizability: Users can easily integrate other feature extractors by implementing a simple function that returns a feature matrix from an image. Convpaint takes care of the user interface, classifier training, data management, and parallelization.

Tiling of images can however interfere with feature extractors like DINOv2 that require whole-image context. If such a feature extractor is selected, these options are disabled by default.

### Quantification and statistical analysis

#### Quantification of segmentation performance

Assessing Convpaint’s performance, especially given its interactive nature, is challenging. Even non-interactive models face problems in unbiased performance evaluation in bioimage analysis.^65^ Given a lack of scribble-annotated datasets, we created an algorithm to generate human-like scribbles from existing ground truth datasets, allowing for an unbiased quantitative assessment of segmentation performance. For each image from the dataset, we generated scribble masks with varying annotation densities. We evaluated three datasets: Cellpose,^1^ FoodSeg103,^39^ and a breast cancer histology slide database^40^ (BCSS). We chose the FoodSeg dataset based on the hypothesis that classical filters would struggle to assign semantic information for items containing highly variable textures. Similarly, we selected the breast cancer dataset, representing a common challenging use case in biological research. The code to automatically generate scribbles and recreate the figures is available on GitHub.^46^ The repository also contains the full results, including multiple performance metrics for each image and classifier at different levels of scribble annotations, as well as plots exploring the effects of feature extractor parameters on segmentation. Tests in Figures 4 and 4; S2A and S2B were run on a GPU workstation (AMD EPYC, nVidia RTX 6000 ADA 48 GB, 256 GB RAM).

#### Dataset preparation

For the BCSS dataset, we applied both automated and manual image selection to ensure annotation quality and computational efficiency. Several images exhibited incomplete or erroneous labels, or showed extreme class imbalances that caused smaller regions to be completely overwritten during automated scribble generation. Specifically, we removed images with incomplete annotations (no pixels labeled as class 0), filtered out images containing only a single class, and excluded windows where any class covered less than 1% of the total area. In addition, five images (AC-A2QJ_1_1_img.png, AC-A7VC_O_2_img.png, AC-A7VC_1_2_img.png, AC-A7VC_2_2_img.png, HN-A2NL_1_1_img.png) were discarded due to clear annotation errors, such as rectangular border regions at image border mislabeled with different class identities. To reduce computation time, we further divided large images into smaller windows. Each window side length was chosen as the largest value ≥1400 px that evenly divides the corresponding image dimension, ensuring consistent coverage without overlap or cropping artifacts. To reduce computation time for the FoodSeg dataset with 4983 images, we excluded images larger than 640 k pixels and used 520 images sampled from the dataset for evaluation.

#### Scribble generation

To closely mimic human annotations, scribbles are created by combining three types of algorithmically generated lines.

1. Center ridge lines: Sampled from the primary skeleton of the ground truth mask.
2. Boundary parallel lines: Sampled from the secondary skeleton, which is derived from the ground truth mask after subtracting the primary skeleton.
3. Boundary perpendicular lines: Lines connecting the primary skeleton to the mask boundary.

By varying the sampling density, we can generate different levels of annotation coverage, such as 0.1% or 1% of the image pixels. The algorithm can also vary scribble type, length, and width, making it versatile for research scenarios beyond the scope of this study, e.g., how scribble types affect segmentation performance. For the Cellpose dataset, which consists mostly of images with numerous small, cell-like objects, we generated many short, 1-pixel-wide scribbles. The ground truth masks were converted from instance segmentation to semantic segmentation (i.e., foreground/background instead of cell IDs). For the FoodSeg dataset, which features fewer but larger regions of different food items, we generated fewer, longer scribbles with a width of 2 pixels. For the histology dataset, we created medium-length scribbles with 2 pixels width.

Refer to Table S1 to see the different configurations and combinations of feature extractors we quantified. In total, we evaluated segmentation performance on 108′642 samples.

### Additional resources

Convpaint installation instructions, documentation, and video tutorials: https://guiwitz.github.io/napari-convpaint/book/Landing.html.

## Supplemental information

Document S1. Figures S1–S4 and Table S1

Data S1. Samples feature extractor performance, related to Figures 4 and 5 Randomly selected images from the Cellpose (Pages 1 and 2), FoodSeg103 (Pages 3 and 4), and BCSS datasets (Pages 5 and 6). For plotting, the scribbles were dilated for better visibility. The number in the lower-right corner of the prediction images shows the mIoU score.

Document S2. Article plus supplemental information
