# Supplementary material for: Convpaint—Interactive pixel classification using pretrained neural networks
Source: Cell Rep Methods. 2026 Mar 16;6(3):101335. doi: 10.1016/j.crmeth.2026.101335 (PMC13030958; doi:10.1016/j.crmeth.2026.101335)
Supplement: Document S1. Figures S1–S4 and Table S1 [file mmc1.pdf]

Cell Reports Methods, Volume *6*

Supplemental information

Convpaint—Interactive pixel classification using pretrained neural networks

Lucien Hinderling, Roman Schwob, Guillaume Witz, Ana Stojiljković, Maciej Dobrzyński, Mykhailo Vladymyrov, Joël Frei, Benjamin Grädel, Agne Frismantiene, and Olivier Pertz

Model	Description	BCSS 0.25%	BCSS 1.0%	BCSS 2.5%	Cellp. 0.25%	Cellp. 1.0%	Cellp. 2.5%	Food. 0.25%	Food. 1.0%	Food. 2.5%
Cellpose	Cellpose backbone	0.36	0.44	0.48	0.72	0.76	0.78	0.38	0.55	0.67
ConvNeXt [S]	ConvNeXt with first convolutional layer; scalings [1, 2]	0.35	0.42	0.45	0.68	0.72	0.74	0.50	0.63	0.73
ConvNeXt [M]	ConvNeXt with convolutional layers [0.0, 1.0.block.0, 1.1.block.0; scalings [1, 2]	0.42	0.54	0.60	0.69	0.74	0.76	0.48	0.65	0.77
EfficientNet [S]	EfficientNet with first convolutional layer; scalings [1, 2]	0.35	0.40	0.43	0.70	0.73	0.75	0.53	0.65	0.73
EfficientNet [M]	EfficientNet with 7 early convolutional layers; scalings [1,2]	0.36	0.43	0.47	0.71	0.75	0.77	0.51	0.65	0.74
Ilastik [S]	Full Ilastik 2D filterset	0.39	0.48	0.52	0.72	0.76	0.78	0.39	0.55	0.67
Ilastik [M]	Full Ilastik 2D filterset; scalings [1, 2, 4]	0.40	0.54	0.62	0.70	0.75	0.78	0.36	0.51	0.67
VGG16 [L1 S2]	VGG16 with convolutional layer [1]; scalings [1, 2]	0.35	0.40	0.42	0.71	0.74	0.75	0.54	0.66	0.74
VGG16 [L1 S3]	VGG16 with convolutional layer [1]; scalings [1, 2, 4]	0.36	0.42	0.45	0.71	0.74	0.76	0.53	0.65	0.74
VGG16 [L1 S5]	VGG16 with convolutional layer [1]; scalings [1, 2, 4, 8, 16]	0.38	0.48	0.54	0.69	0.74	0.77	0.48	0.61	0.73
VGG16 [L3 S2]	VGG16 with convolutional layers [1, 2, 5]; scalings [1, 2]	0.36	0.42	0.46	0.72	0.76	0.77	0.52	0.66	0.75
VGG16 [L3 S3]	VGG16 with convolutional layers [1, 2, 5]; scalings [1, 2, 4]	0.36	0.45	0.50	0.71	0.76	0.78	0.49	0.64	0.75
VGG16 [L3 S5]	VGG16 with convolutional layers [1, 2, 5]; scalings [1, 2, 4, 8, 16]	0.34	0.49	0.59	0.69	0.74	0.77	0.42	0.58	0.73
DINO	DINOv2 using per-patch probabilities	0.40	0.56	0.65	0.58	0.62	0.64	0.58	0.70	0.78
DINO interpolated	DINOv2 with interpolated patch probabilities	0.42	0.58	0.67	0.59	0.64	0.66	0.63	0.74	0.81
DINO Jafar [S]	JAFAR upscaler	0.42	0.57	0.66	0.68	0.72	0.74	0.72	0.83	0.88
DINO Jafar [M]	JAFAR upscaler; scalings [1, 7]	0.42	0.58	0.67	0.69	0.73	0.75	0.71	0.81	0.88
DINO Jafar [L]	JAFAR upscaler; scalings [1, 8, 14]	0.42	0.59	0.68	0.68	0.73	0.75	0.72	0.79	0.87
DINO Jafar [R]	DINO Jafar [S]; image downsampled (2x); seg. smoothing option enabled (2x)	0.43	0.57	0.65	-	-	-	0.69	0.79	0.86
DINO + Gaussian	DINOv2 patches + Gaussian	0.39	0.56	0.65	0.65	0.71	0.74	0.58	0.71	0.80
DINO + Ilastik	DINOv2 patches + Ilastik [S]	0.41	0.58	0.66	0.70	0.75	0.78	0.57	0.71	0.80
DINO + VGG16	DINOv2 patches + VGG16 [L1 S3]	0.40	0.56	0.65	0.70	0.75	0.77	0.61	0.73	0.83
Gaussian	Simple Gaussian filter (sigma = 3)	0.36	0.41	0.42	0.66	0.69	0.70	0.44	0.58	0.67

Table S1. Segmentation performance of different feature extractors (Related to Figures 4,5). The Table lists all models shown in Figures 4 and 5, together with a brief description. Mean mIoU scores are reported for the BCSS (n=538 images), Cellpose (n=540), and FoodSeg (n=520) datasets at annotation densities of 0.25%, 1.0%, and 2.5% of labeled pixels.

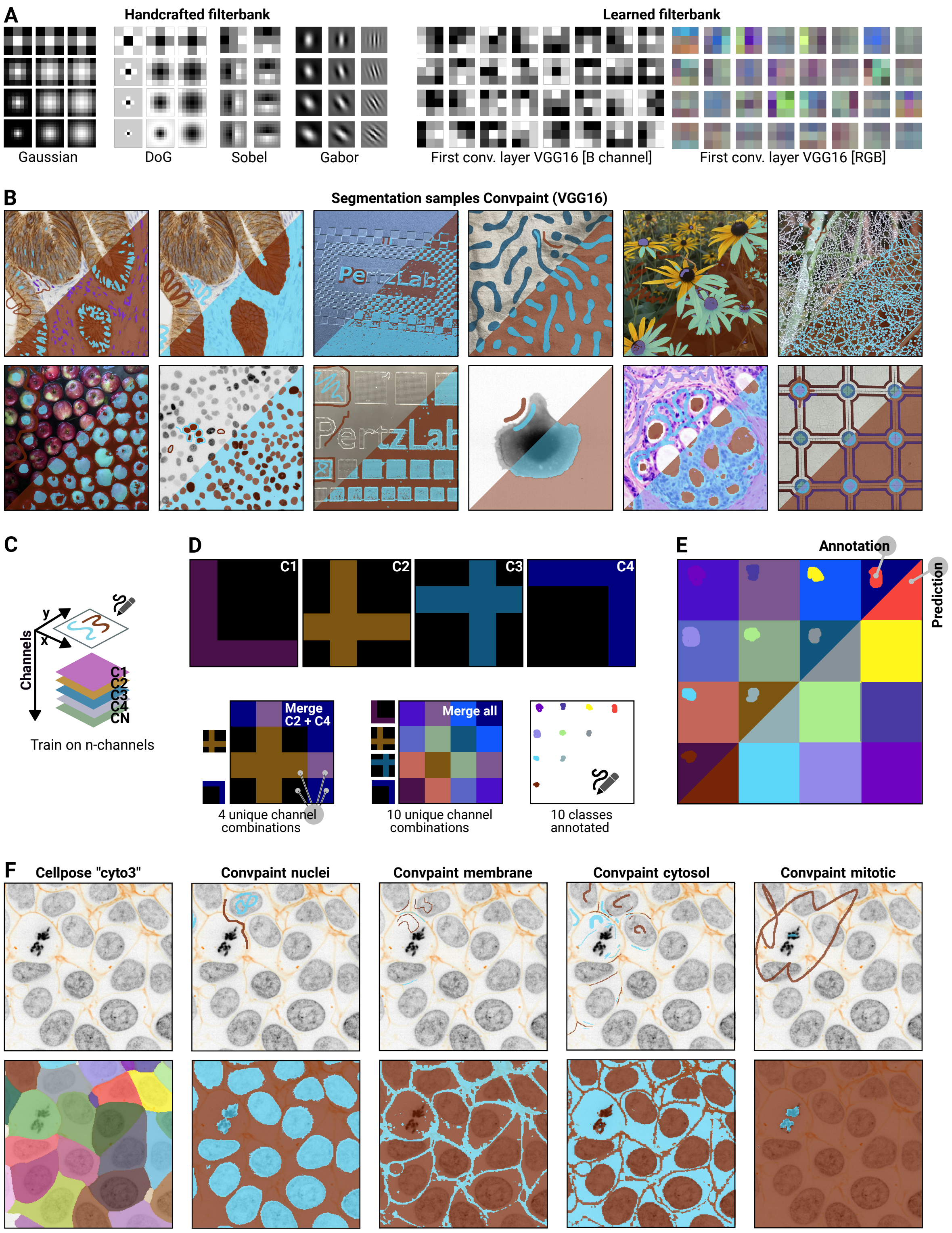

Figure S1. Conceptual overview of the algorithm, example outputs, n-channel compatibility and repurposing feature extractors (Related to Figure 1). (A) Visual comparison of handcrafted vs. learned filters. Left: Filters used classically in handcrafted filter banks. Here we show examples of filter kernels with different parameters for Gaussians, Difference of Gaussians (DoG), Sobel, and Gabor. While these handcrafted filter banks are more interpretable, the patterns they extract often overlap, leading to redundancy among the filters. Right: Filters extracted from the first convolution layer of a CNN (VGG16) network trained for image classification on the ImageNet dataset. The filters have a 3x3x3 shape, which makes them intrinsically capable of extracting correlations between color channels in RGB images. Although VGG16 filters are less interpretable, they are computationally optimized to extract orthogonal image features that are useful for image classification. (B) Image segmentation across diverse domains. All images use VGG16 with the default configuration as feature extractor. Top row, left to right: Two histology images, showing different structures segmented depending on the annotations. A microfabricated structure. Turing pattern printed on fabric. Flowers. Water droplets on a spider net. Bottom row, left to right: Apples. Cells with nuclear marker. Microfabricated structure. Fibroblast cell expressing ERK-KTR biosensor. Histology image. Floor tiles in the University of Bern. (C) Convpaint can extract correlations across an arbitrary number of color input channels. (D) This is demonstrated on an artificial image with four channels, which when merged lead to 10 unique color combinations. These 10 combinations are labeled with 10 class labels that can only be reconstructed if the algorithm considers the interplay of the different channels. (E) Convpaint correctly predicts the correct class label for pixels that were not labeled, with minor artifacts on boundaries between squares. (F) Repurposing Cellpose features: Cellpose is trained to segment whole cells. We can use the intermediate layers of a Cellpose model as input features for Convpaint, enabling to segment other structures by simply annotating them with a few scribbles. Note that if we use this approach, we lose the instance segmentation capabilities of Cellpose.

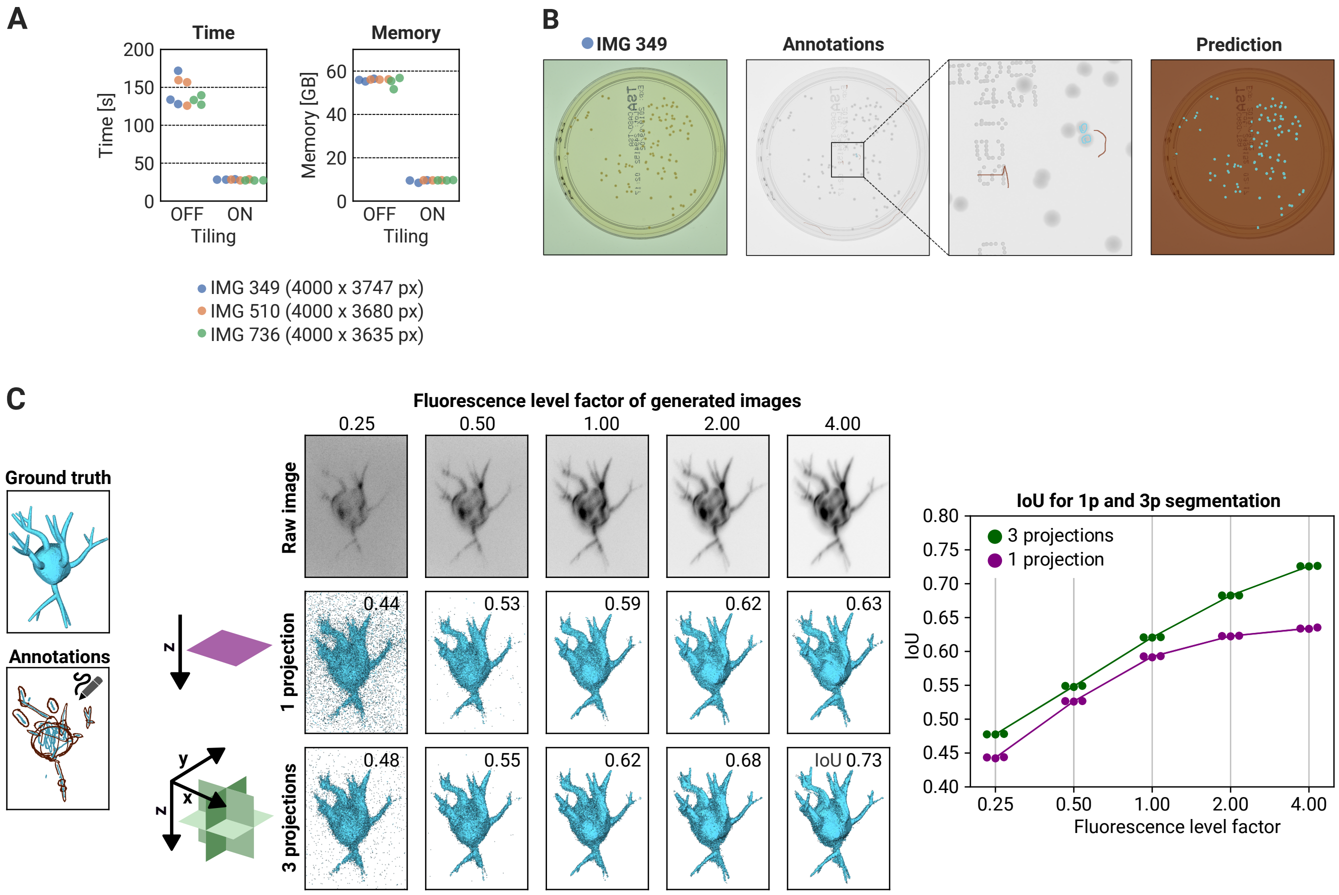

Figure S2. Tiling reduces memory footprint and processing time on large images, using multiple projections improve segmentation performance on 3D data (Related to Figure 1). (A) Three agar plate images (14 MP each) were segmented with and without tiling enabled (three replicates per image). Time includes training + prediction: Tiling OFF 141.72 ± 16.76 s; ON = 27.95 ± 0.66 s. Memory is max. memory usage of system during training + prediction (i.e. including OS and Python runtime): Tiling OFF = 55.54 ± 1.54 GB; ON = 9.47 ± 0.39 GB. (B) Sample showing the input image, annotations, and the corresponding Convpaint segmentation result. (C) Convpaint segmentation performance compared on an artificial cell when extracting features from 1 projection (purple) versus concatenating 3 projections (green), using VGG16 with default configuration as feature extractor. Different signal-to-noise regimes are tested, which are configured by the fluorescence level factor (0.25-4) in the FiloGen software. Performance is measured as intersection over union (IoU). Using 3 projections leads to better segmentation results for all fluorescence level factors. A larger increase in performance is observed for images with a better signal-to-noise ratio.

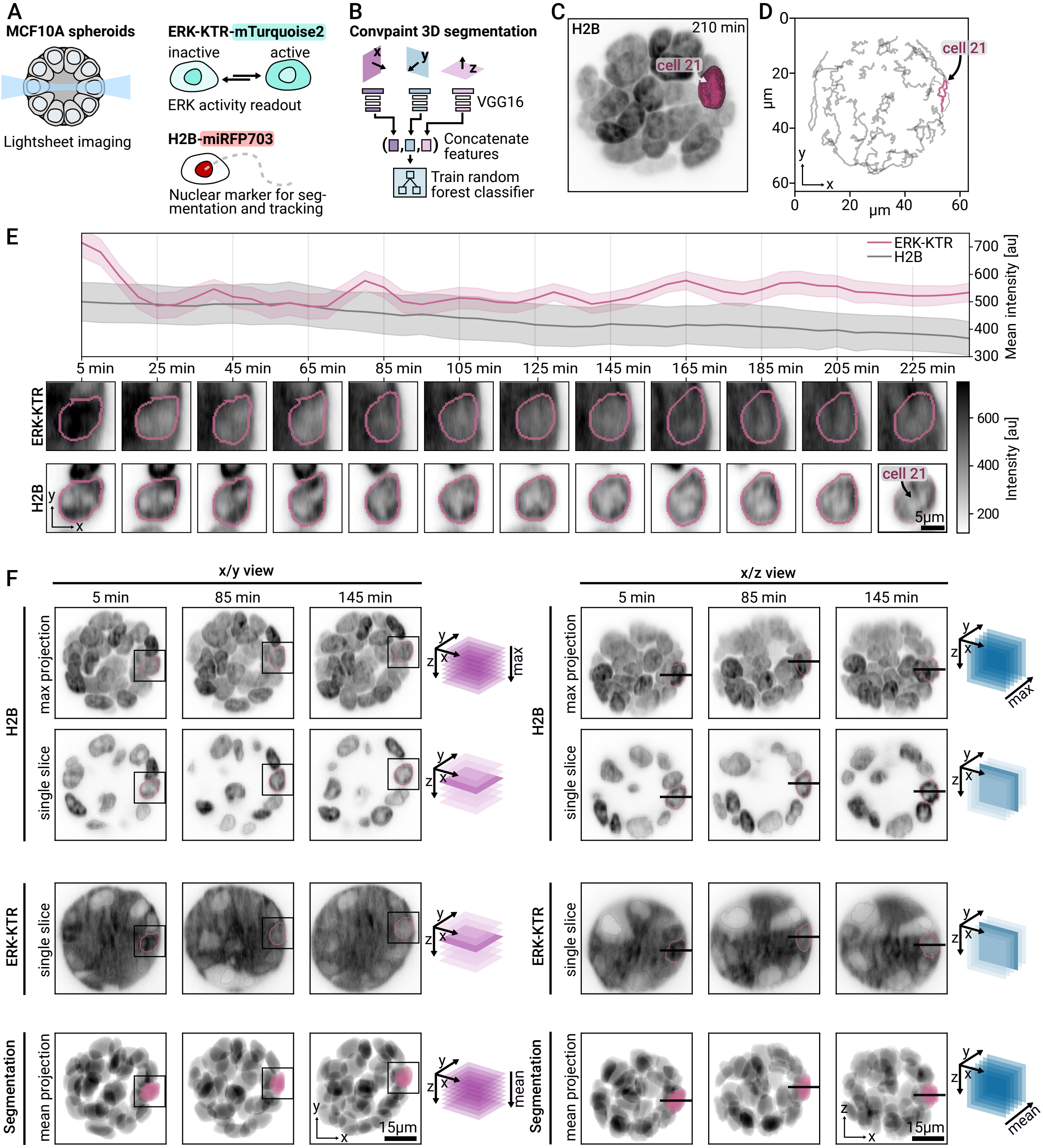

Figure S3. Measuring ERK signaling dynamics at the single-cell level in MCF10A acini (Related to Figure 2). (A) Spheroids are imaged with a lightsheet microscope. The cells express an ERK activity sensor and nuclear marker for segmentation and tracking. (B) Convpaint is used to segment the nuclei in 3D. (C) Panels C-F track a single cell in the spheroid over time, here its mask is shown overlaid on a 3D max projection. (D) Tracks of all cells from 0 to 250 minutes, selected cell highlighted in color. (E) Mean nuclear ERK-KTR intensity over time as a proxy for ERK activity. In comparison, the mean intensity of the nuclear marker shows some bleaching but no fluctuations otherwise. The images show crops around the selected cell (mean of 3 z-slices, [+1,0,-1] around the z position of the cell centroid). Scale bar is 5 µm. (F) Highlighting the tracked position of the cell within the spheroid for different time points, projections, and channels. Box shows insets in panel E. Scale bar is 15 µm.

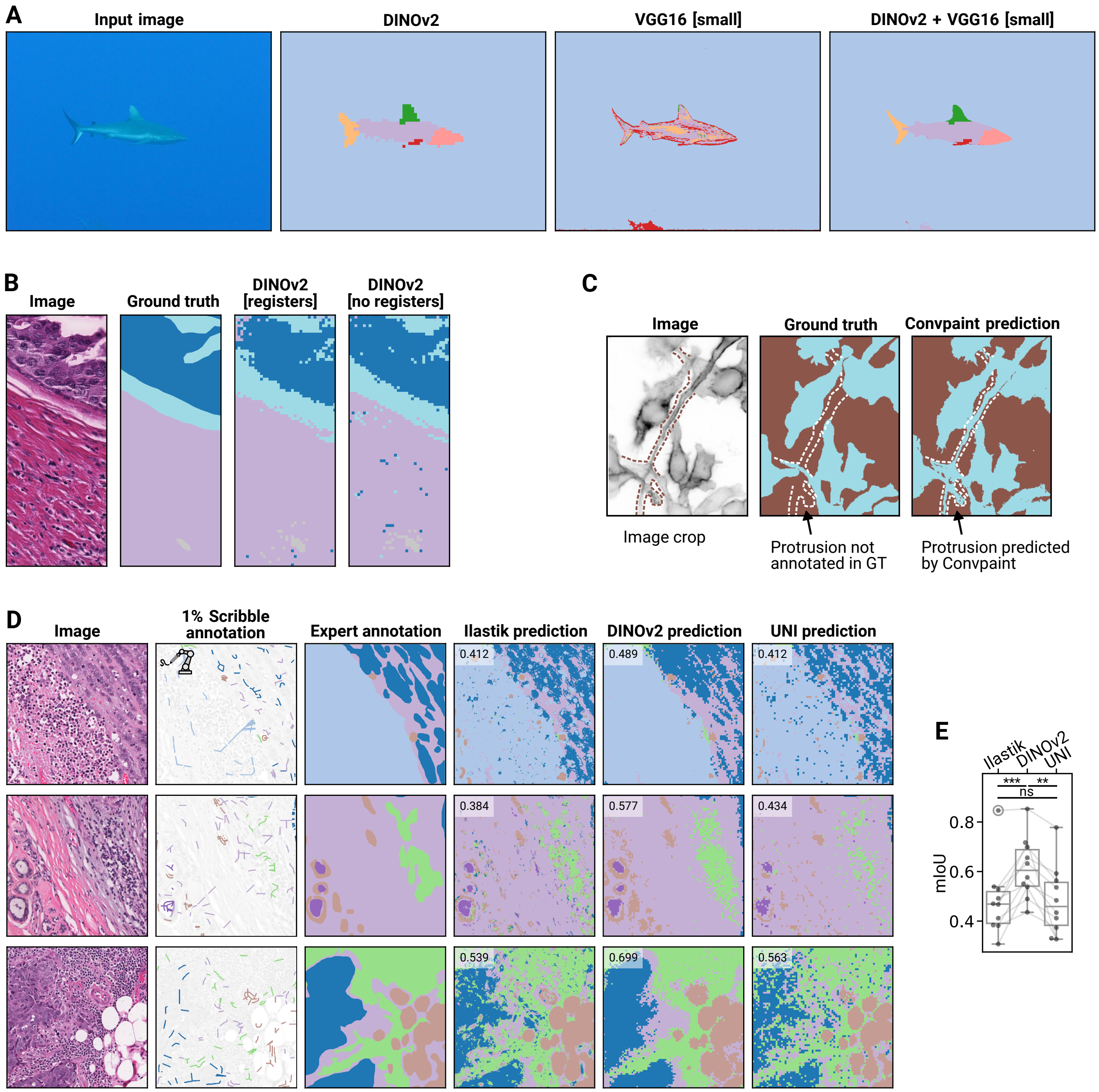

Figure S4. Combining feature extractors and supplementary analysis segmentation performance (Related to Figures 4,5). (A) DINOv2 features excel at capturing abstract semantic information at the patch level, while VGG16 features are good at capturing local spatial information at the pixel level. By concatenating the features of both models, we can leverage the strengths of both models, enhancing spatial precision at mask boundaries while maintaining semantic information. (B) Model performance quantification scores can be limited by the quality of ground-truth annotations. A cell protrusion, missing in the ground truth, is correctly segmented by the model. (C) DINOv2 versions with registers produces predictions with less patch noise (not quantified). (D) Tissue classification performance in histological images, comparing domain specific vs. general vision transformer backbones: From an expert annotation of a histology image, we generated a scribble annotation with 1% image coverage. Convpaint was trained to predict the rest of the image, either using DINOv2, UNI, or a classical filter bank (ilastik) as feature extractor. 3 representative samples shown out of 10 tested images. (E) Overlap scores show significantly better performance of DINOv2 versus classical filter banks (paired t-test $t(9) = 5.04$, $p = 0.0007$) and versus UNI ($t(9) = 4.09$, $p = 0.0027$) on a randomly selected subset of the BCSS dataset (mean mIoU for classical = 0.48, DINOv2 = 0.61, UNI = 0.48). The boxes span from first to third quartile, with a line at the median. Whiskers extend to the farthest point within 1.5x IQR from the box.
