# Supplementary material for: Convpaint—Interactive pixel classification using pretrained neural networks
Source: Cell Rep Methods. 2026 Mar 16;6(3):101335. doi: 10.1016/j.crmeth.2026.101335 (PMC13030958; doi:10.1016/j.crmeth.2026.101335)
Supplement: Data S1. Samples feature extractor performance, related to Figures 4 and 5 — Randomly selected images from the Cellpose (Pages 1 and 2), FoodSeg103 (Pages 3 and 4), and BCSS datasets (Pages 5 and 6). For plotting, the scribbles were dilated for better visibility. The number in the lower-right corner of the prediction images shows the mIoU score. [file mmc2.pdf]

Data S1. Samples feature extractor performance (Related to Figures 4,5). Randomly selected images from the Cellpose (Page 1,2), FoodSeg103 (Page 3,4), and BCSS dataset (Page 5,6). For plotting, the scribbles were dilated for better visibility. The number in the lower-right corner of the prediction images shows the mIoU score.

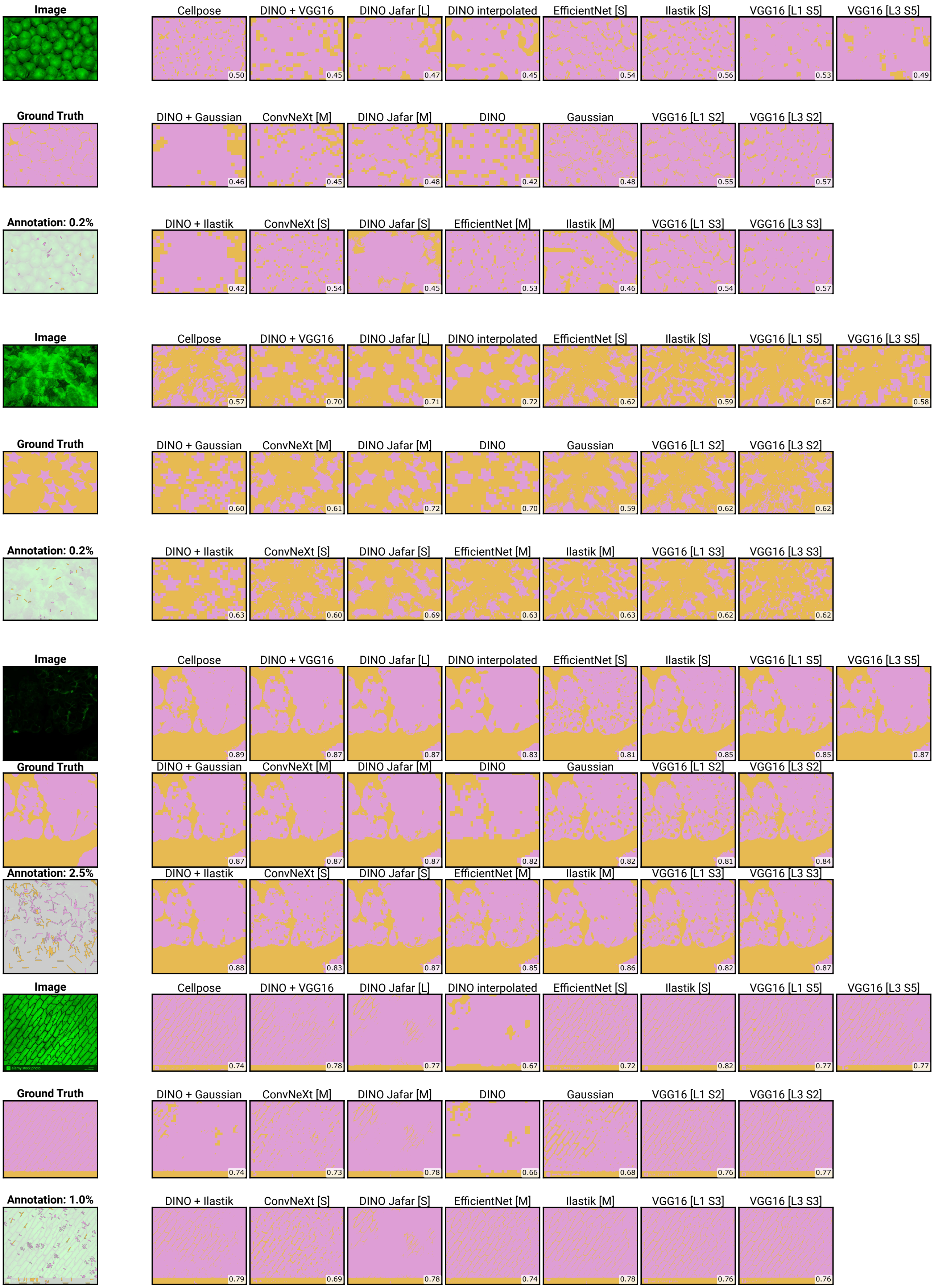

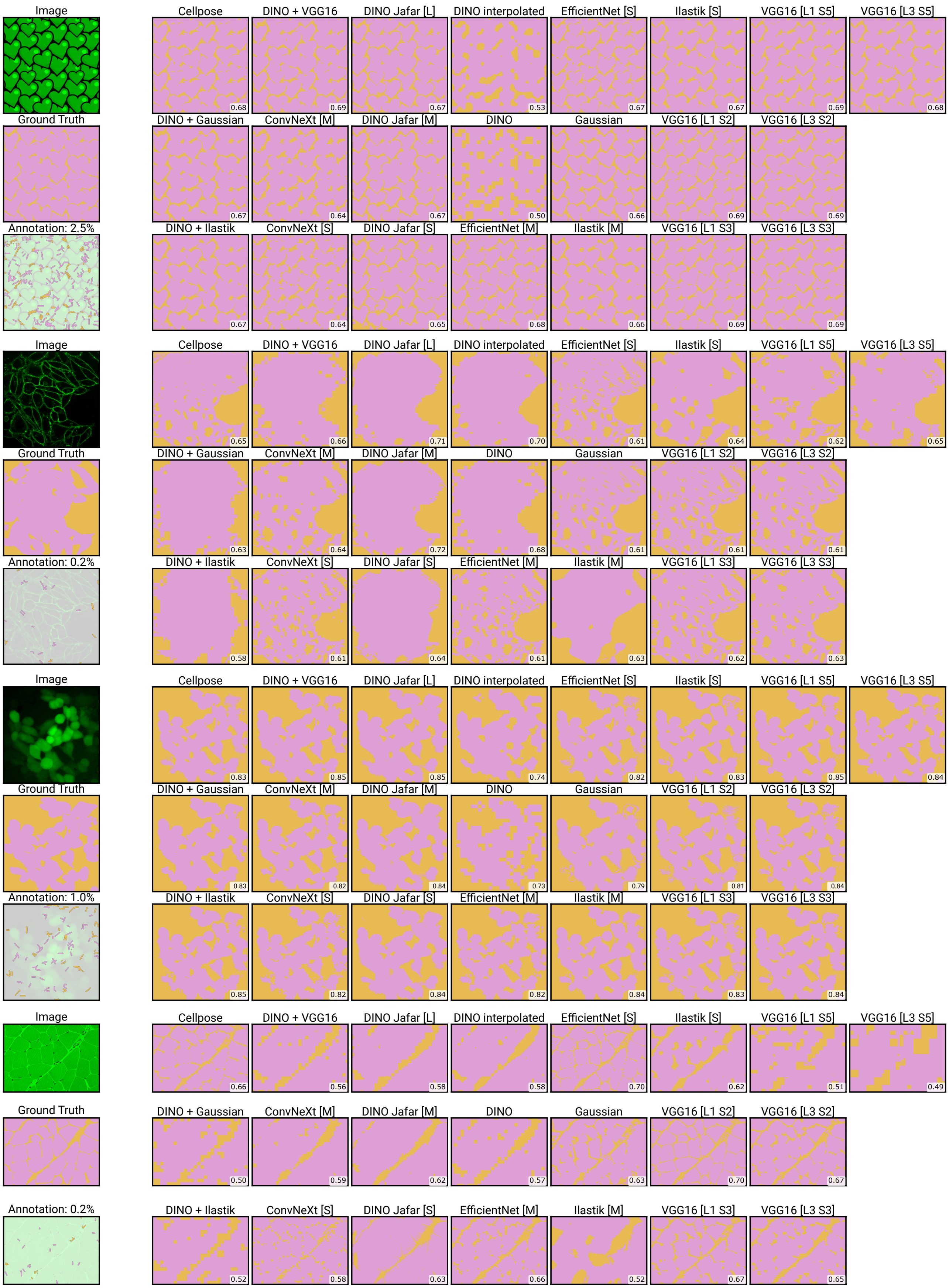
Image
Cellpose
0.68
DINO + VGG16
0.69
DINO Jafar [L]
0.67
DINO interpolated
0.53
EfficientNet [S]
0.67
Ilastik [S]
0.67
VGG16 [L1 S5]
0.69
VGG16 [L3 S5]
0.68
Ground Truth
DINO + Gaussian
0.67
ConvNeXt [M]
0.64
DINO Jafar [M]
0.67
DINO
0.50
Gaussian
0.66
VGG16 [L1 S2]
0.69
VGG16 [L3 S2]
0.69
Annotation: 2.5%
DINO + Ilastik
0.67
ConvNeXt [S]
0.64
DINO Jafar [S]
0.65
EfficientNet [M]
0.68
Ilastik [M]
0.66
VGG16 [L1 S3]
0.69
VGG16 [L3 S3]
0.69
Image
Cellpose
0.65
DINO + VGG16
0.66
DINO Jafar [L]
0.71
DINO interpolated
0.70
EfficientNet [S]
0.61
Ilastik [S]
0.64
VGG16 [L1 S5]
0.62
VGG16 [L3 S5]
0.65
Ground Truth
DINO + Gaussian
0.63
ConvNeXt [M]
0.64
DINO Jafar [M]
0.72
DINO
0.68
Gaussian
0.61
VGG16 [L1 S2]
0.61
VGG16 [L3 S2]
0.61
Annotation: 0.2%
DINO + Ilastik
0.58
ConvNeXt [S]
0.61
DINO Jafar [S]
0.64
EfficientNet [M]
0.61
Ilastik [M]
0.63
VGG16 [L1 S3]
0.62
VGG16 [L3 S3]
0.63
Image
Cellpose
0.83
DINO + VGG16
0.85
DINO Jafar [L]
0.85
DINO interpolated
0.74
EfficientNet [S]
0.82
Ilastik [S]
0.83
VGG16 [L1 S5]
0.85
VGG16 [L3 S5]
0.84
Ground Truth
DINO + Gaussian
0.83
ConvNeXt [M]
0.82
DINO Jafar [M]
0.84
DINO
0.73
Gaussian
0.79
VGG16 [L1 S2]
0.81
VGG16 [L3 S2]
0.84
Annotation: 1.0%
DINO + Ilastik
0.85
ConvNeXt [S]
0.82
DINO Jafar [S]
0.84
EfficientNet [M]
0.82
Ilastik [M]
0.84
VGG16 [L1 S3]
0.83
VGG16 [L3 S3]
0.84
Image
Cellpose
0.66
DINO + VGG16
0.56
DINO Jafar [L]
0.58
DINO interpolated
0.58
EfficientNet [S]
0.70
Ilastik [S]
0.62
VGG16 [L1 S5]
0.51
VGG16 [L3 S5]
0.49
Ground Truth
DINO + Gaussian
0.50
ConvNeXt [M]
0.59
DINO Jafar [M]
0.62
DINO
0.57
Gaussian
0.63
VGG16 [L1 S2]
0.70
VGG16 [L3 S2]
0.67
Annotation: 0.2%
DINO + Ilastik
0.52
ConvNeXt [S]
0.58
DINO Jafar [S]
0.63
EfficientNet [M]
0.66
Ilastik [M]
0.52
VGG16 [L1 S3]
0.67
VGG16 [L3 S3]
0.65

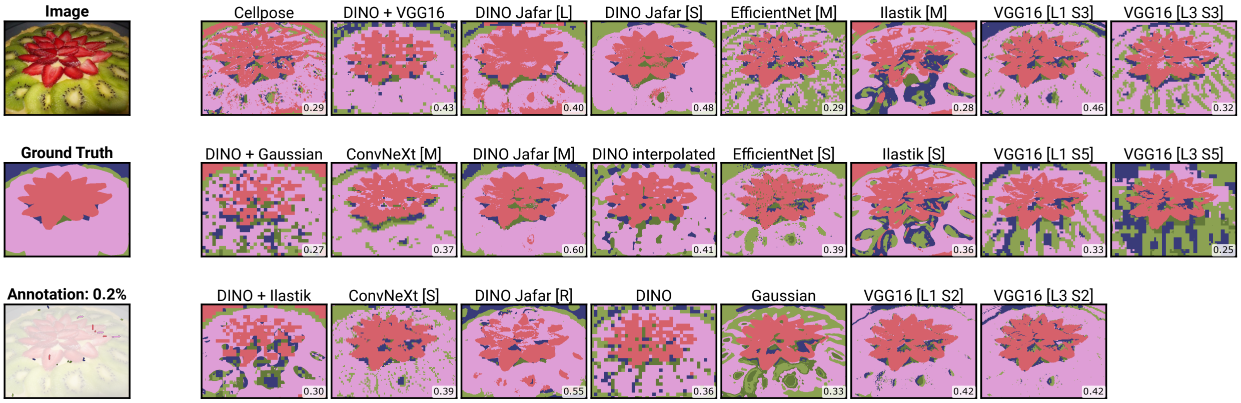
Image
Cellpose
DINO + VGG16
DINO Jafar [L]
DINO Jafar [S]
EfficientNet [M]
Ilastik [M]
VGG16 [L1 S3]
VGG16 [L3 S3]
Ground Truth
DINO + Gaussian
ConvNeXt [M]
DINO Jafar [M]
DINO interpolated
EfficientNet [S]
Ilastik [S]
VGG16 [L1 S5]
VGG16 [L3 S5]
Annotation: 0.2%
DINO + Ilastik
ConvNeXt [S]
DINO Jafar [R]
DINO
Gaussian
VGG16 [L1 S2]
VGG16 [L3 S2]

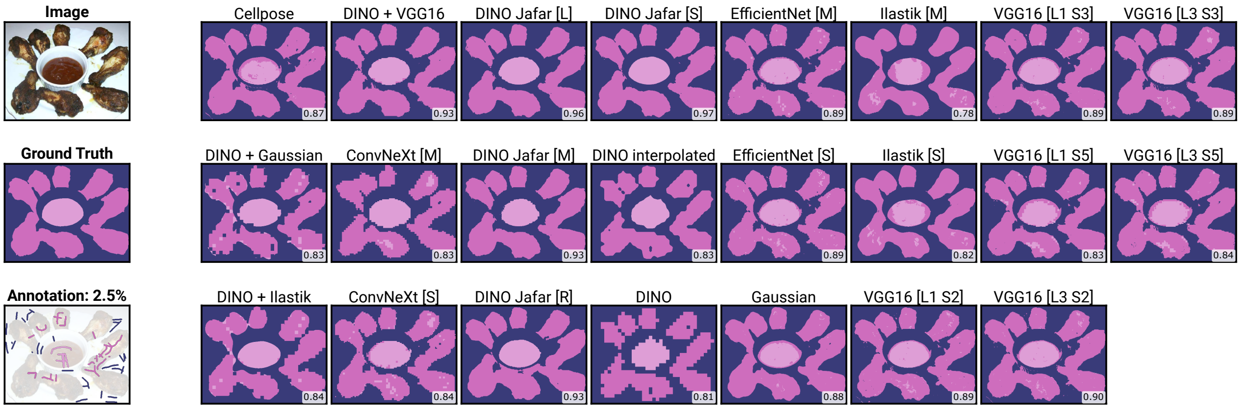
Image
Cellpose
DINO + VGG16
DINO Jafar [L]
DINO Jafar [S]
EfficientNet [M]
Ilastik [M]
VGG16 [L1 S3]
VGG16 [L3 S3]
Ground Truth
DINO + Gaussian
ConvNeXt [M]
DINO Jafar [M]
DINO interpolated
EfficientNet [S]
Ilastik [S]
VGG16 [L1 S5]
VGG16 [L3 S5]
Annotation: 2.5%
DINO + Ilastik
ConvNeXt [S]
DINO Jafar [R]
DINO
Gaussian
VGG16 [L1 S2]
VGG16 [L3 S2]

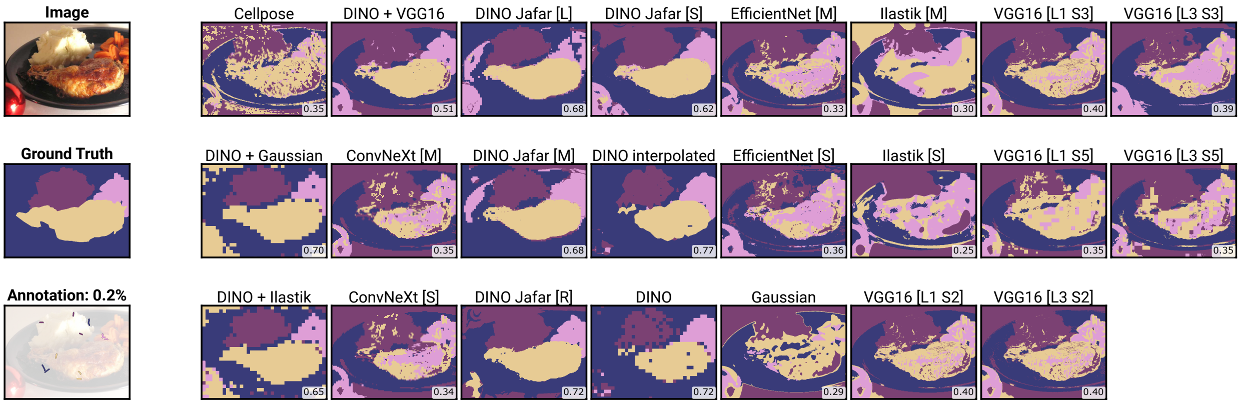
Image
Cellpose
DINO + VGG16
DINO Jafar [L]
DINO Jafar [S]
EfficientNet [M]
Ilastik [M]
VGG16 [L1 S3]
VGG16 [L3 S3]
Ground Truth
DINO + Gaussian
ConvNeXt [M]
DINO Jafar [M]
DINO interpolated
EfficientNet [S]
Ilastik [S]
VGG16 [L1 S5]
VGG16 [L3 S5]
Annotation: 0.2%
DINO + Ilastik
ConvNeXt [S]
DINO Jafar [R]
DINO
Gaussian
VGG16 [L1 S2]
VGG16 [L3 S2]

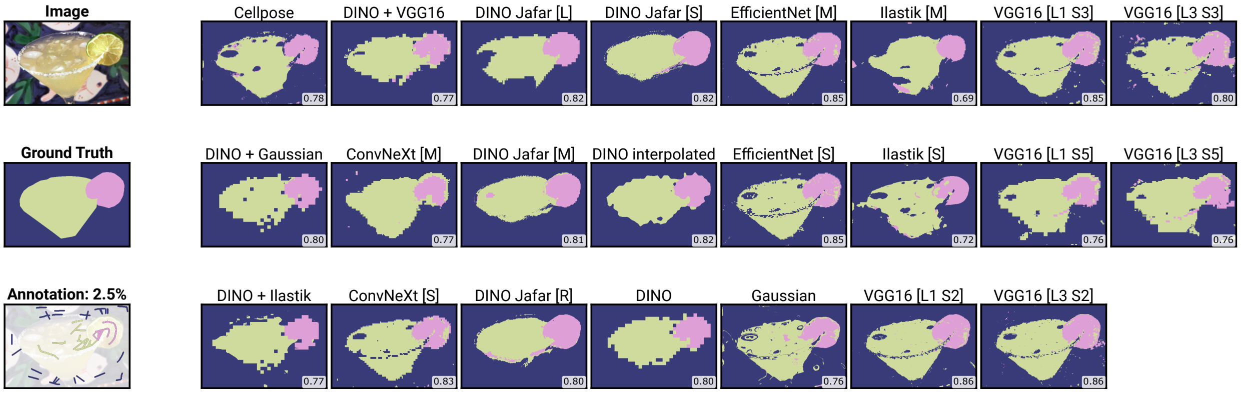
Image
Cellpose
DINO + VGG16
DINO Jafar [L]
DINO Jafar [S]
EfficientNet [M]
Ilastik [M]
VGG16 [L1 S3]
VGG16 [L3 S3]
Ground Truth
DINO + Gaussian
ConvNeXt [M]
DINO Jafar [M]
DINO interpolated
EfficientNet [S]
Ilastik [S]
VGG16 [L1 S5]
VGG16 [L3 S5]
Annotation: 2.5%
DINO + Ilastik
ConvNeXt [S]
DINO Jafar [R]
DINO
Gaussian
VGG16 [L1 S2]
VGG16 [L3 S2]

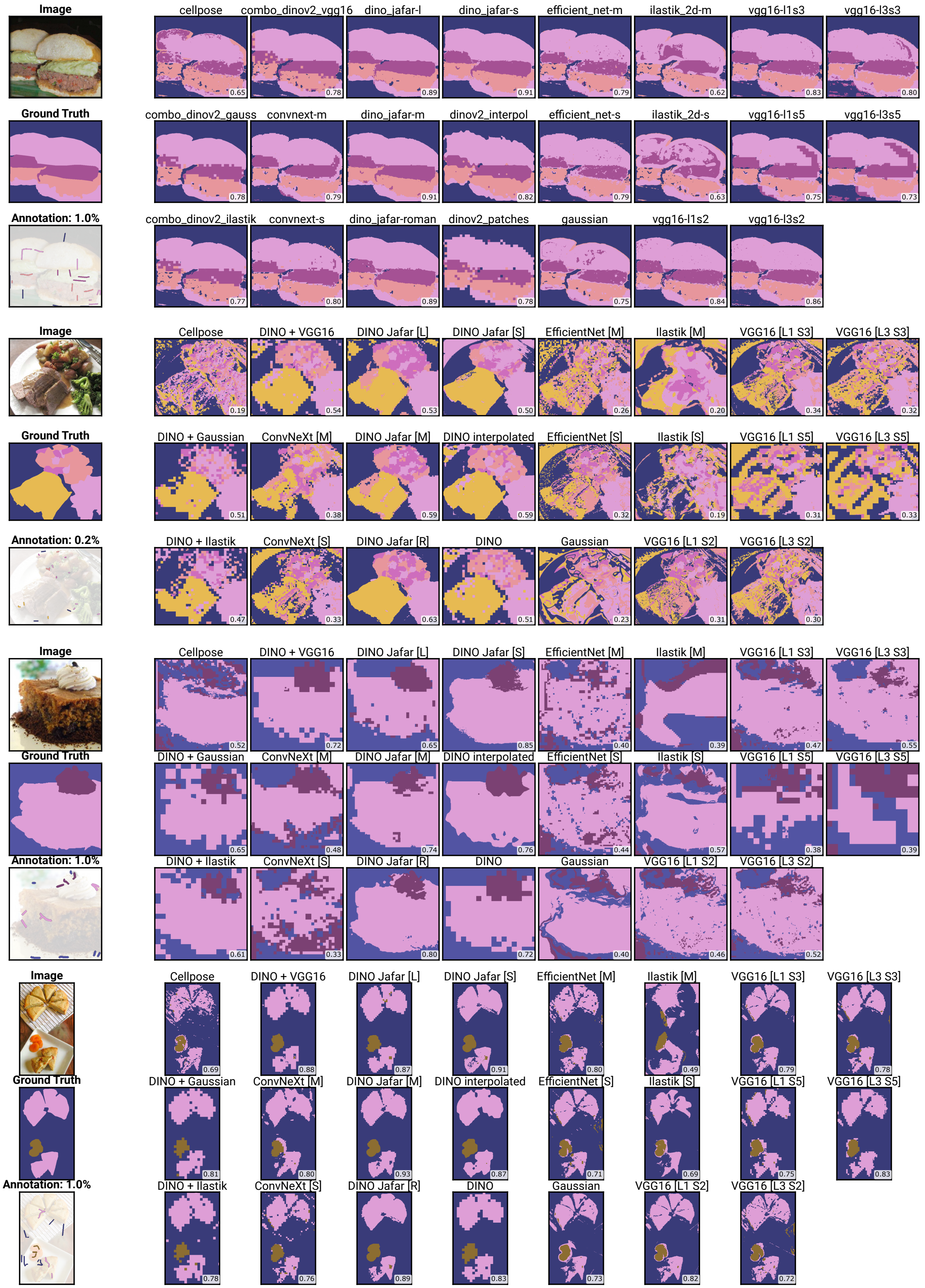
Image
cellpose 0.65
combo_dinov2_vgg16 0.78
dino_jafar-l 0.89
dino_jafar-s 0.91
efficient_net-m 0.79
ilastik_2d-m 0.62
vgg16-l1s3 0.83
vgg16-l3s3 0.80
Ground Truth
combo_dinov2_gauss 0.78
convnext-m 0.79
dino_jafar-m 0.91
dinov2_interpol 0.82
efficient_net-s 0.79
ilastik_2d-s 0.63
vgg16-l1s5 0.75
vgg16-l3s5 0.73
Annotation: 1.0%
combo_dinov2_ilastik 0.77
convnext-s 0.80
dino_jafar-roman 0.89
dinov2_patches 0.78
gaussian 0.75
vgg16-l1s2 0.84
vgg16-l3s2 0.86
Image
Cellpose 0.19
DINO + VGG16 0.54
DINO Jafar [L] 0.53
DINO Jafar [S] 0.50
EfficientNet [M] 0.26
Ilastik [M] 0.20
VGG16 [L1 S3] 0.34
VGG16 [L3 S3] 0.32
Ground Truth
DINO + Gaussian 0.51
ConvNeXt [M] 0.38
DINO Jafar [M] 0.59
DINO interpolated 0.59
EfficientNet [S] 0.32
Ilastik [S] 0.19
VGG16 [L1 S5] 0.31
VGG16 [L3 S5] 0.33
Annotation: 0.2%
DINO + Ilastik 0.47
ConvNeXt [S] 0.33
DINO Jafar [R] 0.63
DINO 0.51
Gaussian 0.23
VGG16 [L1 S2] 0.31
VGG16 [L3 S2] 0.30
Image
Cellpose 0.52
DINO + VGG16 0.72
DINO Jafar [L] 0.65
DINO Jafar [S] 0.85
EfficientNet [M] 0.40
Ilastik [M] 0.39
VGG16 [L1 S3] 0.47
VGG16 [L3 S3] 0.55
Ground Truth
DINO + Gaussian 0.65
ConvNeXt [M] 0.48
DINO Jafar [M] 0.74
DINO interpolated 0.76
EfficientNet [S] 0.44
Ilastik [S] 0.57
VGG16 [L1 S5] 0.38
VGG16 [L3 S5] 0.39
Annotation: 1.0%
DINO + Ilastik 0.61
ConvNeXt [S] 0.33
DINO Jafar [R] 0.80
DINO 0.72
Gaussian 0.40
VGG16 [L1 S2] 0.46
VGG16 [L3 S2] 0.52
Image
Cellpose 0.69
DINO + VGG16 0.88
DINO Jafar [L] 0.87
DINO Jafar [S] 0.91
EfficientNet [M] 0.80
Ilastik [M] 0.49
VGG16 [L1 S3] 0.79
VGG16 [L3 S3] 0.78
Ground Truth
DINO + Gaussian 0.81
ConvNeXt [M] 0.80
DINO Jafar [M] 0.93
DINO interpolated 0.87
EfficientNet [S] 0.71
Ilastik [S] 0.69
VGG16 [L1 S5] 0.75
VGG16 [L3 S5] 0.83
Annotation: 1.0%
DINO + Ilastik 0.78
ConvNeXt [S] 0.76
DINO Jafar [R] 0.89
DINO 0.83
Gaussian 0.73
VGG16 [L1 S2] 0.82
VGG16 [L3 S2] 0.72

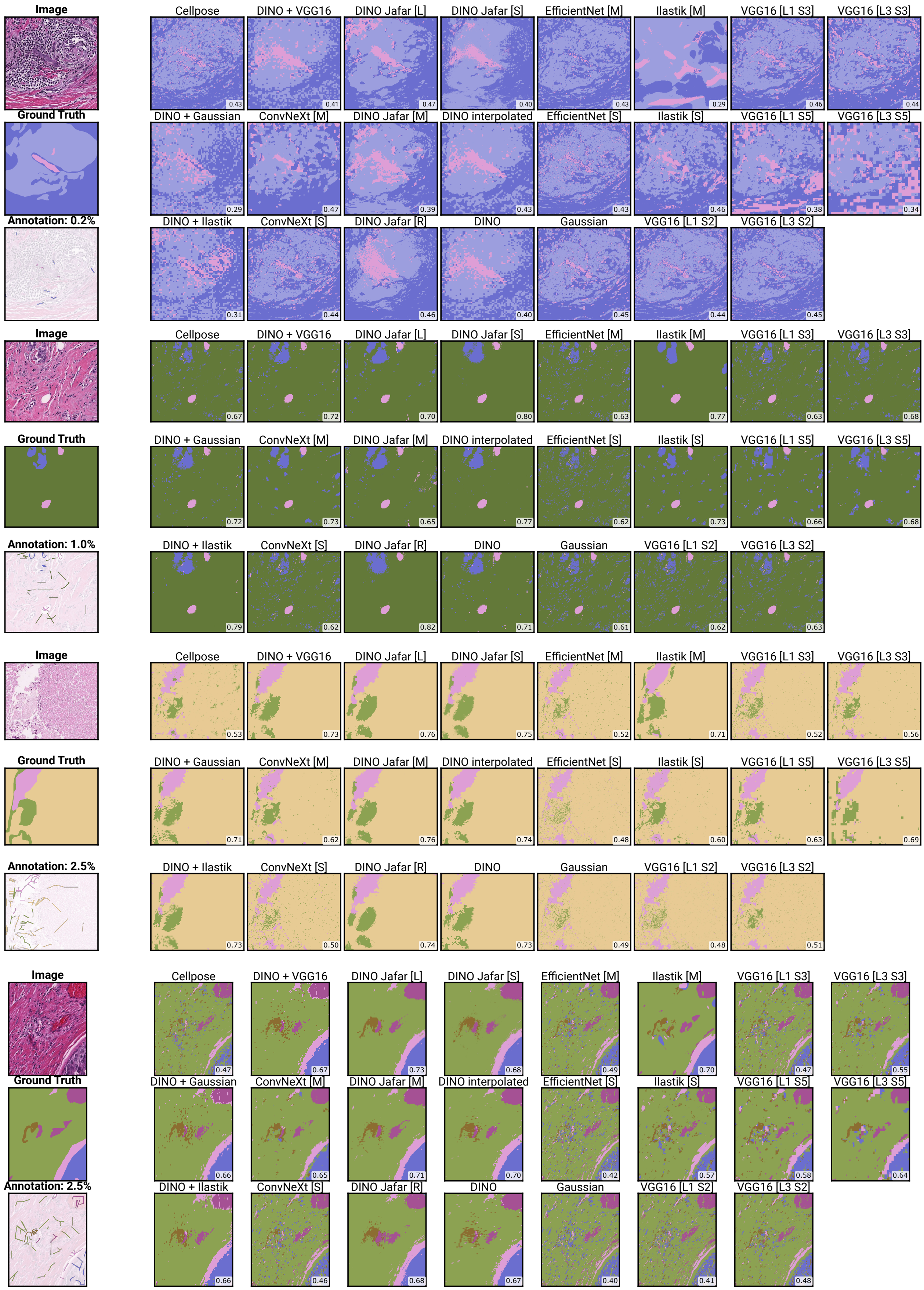
Image
Cellpose 0.43
DINO + VGG16 0.41
DINO Jafar [L] 0.47
DINO Jafar [S] 0.40
EfficientNet [M] 0.43
Ilastik [M] 0.29
VGG16 [L1 S3] 0.46
VGG16 [L3 S3] 0.44
Ground Truth
DINO + Gaussian 0.29
ConvNeXt [M] 0.47
DINO Jafar [M] 0.39
DINO interpolated 0.43
EfficientNet [S] 0.43
Ilastik [S] 0.46
VGG16 [L1 S5] 0.38
VGG16 [L3 S5] 0.34
Annotation: 0.2%
DINO + Ilastik 0.31
ConvNeXt [S] 0.44
DINO Jafar [R] 0.46
DINO 0.40
Gaussian 0.45
VGG16 [L1 S2] 0.44
VGG16 [L3 S2] 0.45
Image
Cellpose 0.67
DINO + VGG16 0.72
DINO Jafar [L] 0.70
DINO Jafar [S] 0.80
EfficientNet [M] 0.63
Ilastik [M] 0.77
VGG16 [L1 S3] 0.63
VGG16 [L3 S3] 0.68
Ground Truth
DINO + Gaussian 0.72
ConvNeXt [M] 0.73
DINO Jafar [M] 0.65
DINO interpolated 0.77
EfficientNet [S] 0.62
Ilastik [S] 0.73
VGG16 [L1 S5] 0.66
VGG16 [L3 S5] 0.68
Annotation: 1.0%
DINO + Ilastik 0.79
ConvNeXt [S] 0.62
DINO Jafar [R] 0.82
DINO 0.71
Gaussian 0.61
VGG16 [L1 S2] 0.62
VGG16 [L3 S2] 0.63
Image
Cellpose 0.53
DINO + VGG16 0.73
DINO Jafar [L] 0.76
DINO Jafar [S] 0.75
EfficientNet [M] 0.52
Ilastik [M] 0.71
VGG16 [L1 S3] 0.52
VGG16 [L3 S3] 0.56
Ground Truth
DINO + Gaussian 0.71
ConvNeXt [M] 0.62
DINO Jafar [M] 0.76
DINO interpolated 0.74
EfficientNet [S] 0.48
Ilastik [S] 0.60
VGG16 [L1 S5] 0.63
VGG16 [L3 S5] 0.69
Annotation: 2.5%
DINO + Ilastik 0.73
ConvNeXt [S] 0.50
DINO Jafar [R] 0.74
DINO 0.73
Gaussian 0.49
VGG16 [L1 S2] 0.48
VGG16 [L3 S2] 0.51
Image
Cellpose 0.47
DINO + VGG16 0.67
DINO Jafar [L] 0.73
DINO Jafar [S] 0.68
EfficientNet [M] 0.49
Ilastik [M] 0.70
VGG16 [L1 S3] 0.47
VGG16 [L3 S3] 0.55
Ground Truth
DINO + Gaussian 0.66
ConvNeXt [M] 0.65
DINO Jafar [M] 0.71
DINO interpolated 0.70
EfficientNet [S] 0.42
Ilastik [S] 0.57
VGG16 [L1 S5] 0.58
VGG16 [L3 S5] 0.64
Annotation: 2.5%
DINO + Ilastik 0.66
ConvNeXt [S] 0.46
DINO Jafar [R] 0.68
DINO 0.67
Gaussian 0.40
VGG16 [L1 S2] 0.41
VGG16 [L3 S2] 0.48

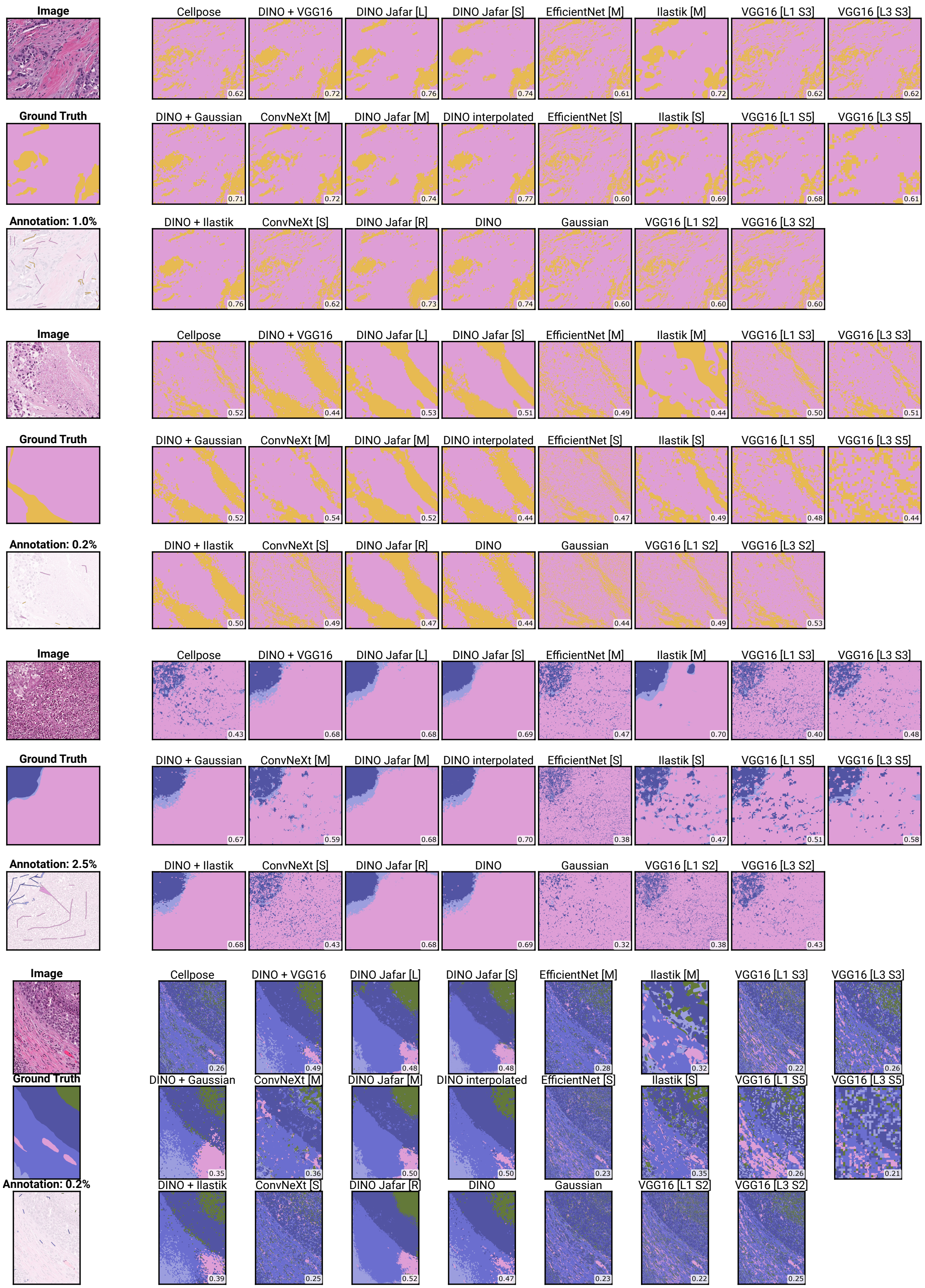
Image
Cellpose 0.62
DINO + VGG16 0.72
DINO Jafar [L] 0.76
DINO Jafar [S] 0.74
EfficientNet [M] 0.61
Ilastik [M] 0.72
VGG16 [L1 S3] 0.62
VGG16 [L3 S3] 0.62
Ground Truth
DINO + Gaussian 0.71
ConvNeXt [M] 0.72
DINO Jafar [M] 0.74
DINO interpolated 0.77
EfficientNet [S] 0.60
Ilastik [S] 0.69
VGG16 [L1 S5] 0.68
VGG16 [L3 S5] 0.61
Annotation: 1.0%
DINO + Ilastik 0.76
ConvNeXt [S] 0.62
DINO Jafar [R] 0.73
DINO 0.74
Gaussian 0.60
VGG16 [L1 S2] 0.60
VGG16 [L3 S2] 0.60
Image
Cellpose 0.52
DINO + VGG16 0.44
DINO Jafar [L] 0.53
DINO Jafar [S] 0.51
EfficientNet [M] 0.49
Ilastik [M] 0.44
VGG16 [L1 S3] 0.50
VGG16 [L3 S3] 0.51
Ground Truth
DINO + Gaussian 0.52
ConvNeXt [M] 0.54
DINO Jafar [M] 0.52
DINO interpolated 0.44
EfficientNet [S] 0.47
Ilastik [S] 0.49
VGG16 [L1 S5] 0.48
VGG16 [L3 S5] 0.44
Annotation: 0.2%
DINO + Ilastik 0.50
ConvNeXt [S] 0.49
DINO Jafar [R] 0.47
DINO 0.44
Gaussian 0.44
VGG16 [L1 S2] 0.49
VGG16 [L3 S2] 0.53
Image
Cellpose 0.43
DINO + VGG16 0.68
DINO Jafar [L] 0.68
DINO Jafar [S] 0.69
EfficientNet [M] 0.47
Ilastik [M] 0.70
VGG16 [L1 S3] 0.40
VGG16 [L3 S3] 0.48
Ground Truth
DINO + Gaussian 0.67
ConvNeXt [M] 0.59
DINO Jafar [M] 0.68
DINO interpolated 0.70
EfficientNet [S] 0.38
Ilastik [S] 0.47
VGG16 [L1 S5] 0.51
VGG16 [L3 S5] 0.58
Annotation: 2.5%
DINO + Ilastik 0.68
ConvNeXt [S] 0.43
DINO Jafar [R] 0.68
DINO 0.69
Gaussian 0.32
VGG16 [L1 S2] 0.38
VGG16 [L3 S2] 0.43
Image
Cellpose 0.26
DINO + VGG16 0.49
DINO Jafar [L] 0.48
DINO Jafar [S] 0.48
EfficientNet [M] 0.28
Ilastik [M] 0.32
VGG16 [L1 S3] 0.22
VGG16 [L3 S3] 0.26
Ground Truth
DINO + Gaussian 0.35
ConvNeXt [M] 0.36
DINO Jafar [M] 0.50
DINO interpolated 0.50
EfficientNet [S] 0.23
Ilastik [S] 0.35
VGG16 [L1 S5] 0.26
VGG16 [L3 S5] 0.21
Annotation: 0.2%
DINO + Ilastik 0.39
ConvNeXt [S] 0.25
DINO Jafar [R] 0.52
DINO 0.47
Gaussian 0.23
VGG16 [L1 S2] 0.22
VGG16 [L3 S2] 0.25
